# Spatiotemporal Monitoring of Cropland Soil Organic Carbon Changes From Space

**DOI:** 10.1111/gcb.17608

**Published:** 2024-12-09

**Authors:** Tom Broeg, Axel Don, Martin Wiesmeier, Thomas Scholten, Stefan Erasmi

**Affiliations:** ^1^ Thünen Earth Observation (ThEO) Thünen Institute of Farm Economics Braunschweig Germany; ^2^ Department of Geosciences, Soil Science and Geomorphology University of Tübingen Tübingen Germany; ^3^ Thünen Institute of Climate‐Smart Agriculture Braunschweig Germany; ^4^ Bavarian State Research Center for Agriculture Institute for Agroecology and Organic Farming Freising Germany

**Keywords:** bare soil, carbon sequestration, change detection, climate change, earth observation, remote sensing, soil reflectance composite, space–time model

## Abstract

Soil monitoring requires accurate and spatially explicit information on soil organic carbon (SOC) trends and changes over time. Spatiotemporal SOC models based on Earth Observation (EO) satellite data can support large‐scale SOC monitoring but often lack sufficient temporal validation based on long‐term soil data. In this study, we used repeated SOC samples from 1986 to 2022 and a time series of multispectral bare soil observations (Landsat and Sentinel‐2) to model high‐resolution cropland SOC trends for almost four decades. An in‐depth validation of the temporal model uncertainty and accuracy of the derived SOC trends was conducted based on a network of 100 long‐term monitoring sites that were continuously resampled every 5 years. While the general SOC prediction accuracy was high (*R* ^2^ = 0.61; RMSE = 5.6 g kg^−1^), the direct validation of the derived SOC trends revealed a significantly greater uncertainty (*R* ^2^ = 0.16; *p* < 0.0001), even though predicted and measured values showed similar distributions. Classifying the results into declining and increasing SOC trends, we found that 95% of all sites were either correctly identified or predicted as stable (*p* < 0.001), highlighting the potential of our findings. Increased accuracies for SOC trends were found in soils with higher SOC contents (*R* ^2^ = 0.4) and sites with reduced tillage (*R* ^2^ = 0.26). Based on the signal‐to‐noise ratio and temporal model uncertainty, we were able to show that the necessary time frame to detect SOC trends strongly depends on the absolute SOC changes present in the soils. Our findings highlight the potential to generate significant cropland SOC trend maps based on EO data and underline the necessity for direct validation with repeated soil samples and long‐term SOC measurements. This study marks an important step toward the usability and integration of EO‐based SOC maps for large‐scale soil carbon monitoring.

## Graphical abstract

This study explores how satellite data and soil samples can track changes in soil organic carbon (SOC) over time, which is vital for understanding soil health and supporting climate‐smart agriculture. Using nearly 40 years of satellite imagery and soil samples from 100 long‐term monitoring sites, we modeled SOC trends in croplands and found that the uncertainty is much higher in comparison to the general prediction accuracy. While our results underline the promise of satellite‐based SOC maps for large‐scale soil monitoring, they also stress the need for validation with real soil data.

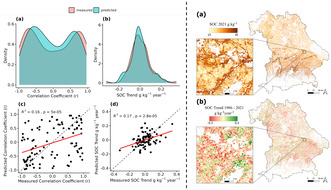

## 1 Introduction

Soils are an essential part of the global carbon cycle and could play an integral role in our mission toward climate neutrality (Amelung et al. 2020). Policies, such as the European Green Deal, heavily rely on sustainable soil management to achieve comprehensive goals regarding soil health, climate protection, and biodiversity (Montanarella and Panagos 2021). In this context, it is increasingly important to monitor and report the status of agricultural soils. Advanced soil monitoring systems are needed to track long‐term changes and to inform about sustainable management and greenhouse gas emissions (Smith et al. 2020). Soil organic carbon (SOC) is one of the key indicators for soil health and carbon sequestration and has been the focus of current soil monitoring strategies (Lal 2016). It has been shown that changes in SOC are closely related to agricultural practices (Paustian et al. 2016). Hence, the global demand for fine‐scale information on SOC dynamics and management data of agricultural land is increasing (Paustian et al. 2019). Accurate information on long‐term SOC changes can be used to verify and track the effects of soil management and help quantify carbon sequestration in carbon farming schemes (European Commission 2022).

Earth Observation (EO) provides extensive information on land cover and land use of agricultural soils. Starting with the launch of Landsat 5 in 1984, the availability of multispectral satellite data has increased substantially during the last decades. The potential of remote sensing to predict SOC dynamics was already recognized more than a decade ago (Croft, Kuhn, and Anderson 2012). Multitemporal soil samples can be combined with the corresponding EO data to conduct spatiotemporal models and generate information on SOC trends and changes (Hengl et al. 2018; Heuvelink et al. 2021). Based on this, spatially explicit and fine‐scale SOC predictions can act as an important resource to support existing soil monitoring programs such as the Land Use/Cover Area Frame Survey (LUCAS) soil survey in Europe (De Rosa et al. 2024). However, this potential has rarely been realized due to challenges with SOC changes being small (< 0.5 g kg^−1^ year^−1^), the lack of appropriate ground truth data from long‐term soil monitoring, and the low availability of EO data for early years (Croft, Kuhn, and Anderson 2012).

Recent studies have shown that spectral information of bare soils can be used to compute high‐resolution, area‐wide SOC maps (Broeg, Don, Gocht, et al. 2024; Möller et al. 2022; Zepp et al. 2021). Soil reflectance composites (SRC) have been generated by averaging bare soil observations over time to obtain the mean soil reflectance at the pixel level (Demattê et al. 2018; Heiden et al. 2022; Rogge et al. 2018). Despite improving the quality of static SOC maps (Broeg et al. 2023), SRC have been rarely used to predict dynamic SOC trends and changes. This is because SRC are usually generated with data from multiple years to increase the number of bare soil observations and to reduce the noise of the soil signal (Zepp et al. 2023). Instead, several studies rely on composites of the full EO time series to predict SOC changes (Meng et al. 2024; Paul et al. 2020; Venter et al. 2021; Yang et al. 2023). In this case, however, the presence of vegetation can significantly affect the spectral SOC signal in the composites (Dvorakova et al. 2022).

Several studies have raised concerns about the high uncertainty and low signal‐to‐noise ratio of EO‐based SOC models (Chabrillat et al. 2019; Croft, Kuhn, and Anderson 2012). For spatiotemporal SOC models and the prediction of SOC trends, the signal can be defined as the absolute SOC change in time, while the noise describes the SOC prediction uncertainty in space (Heuvelink et al. 2021; Paustian et al. 2019). In this regard, the high local variability of SOC represents a major challenge to detect SOC trends (Poeplau, Prietz, and Don 2022). Predicting SOC stocks in Argentina from 1982 to 2017, Heuvelink et al. (2021) were able to demonstrate that the spatial SOC variation can be up to one order of magnitude higher than the temporal one. This is especially true for cropland soils, as SOC contents are usually much smaller compared to other land‐use types, and it can take up to 15 years to measure significant changes (Poeplau and Don 2013; Smith 2004). It therefore remains crucial to evaluate the signal‐to‐noise ratio and the conditions under which spatiotemporal models can predict SOC trends in cropland soils.

Soil data from resampled sites is necessary to directly validate predicted SOC changes and to test the temporal accuracy of spatiotemporal models in an unbiased manner. This is, however, not feasible in most regions due to the low availability of SOC samples from long‐term monitoring programs. Meng et al. (2024) used Landsat and soil data from 1984 to 2021 to predict large‐scale SOC time series but had to rely on indirect measures to estimate the temporal model accuracy due to limited reference data. For the same reason, Venter et al. (2021) were unable to validate the SOC trend map that was produced for South Africa using Landsat composites and yearly SOC predictions from 1986 to 2019. Because of missing data on SOC change and the lack of direct validation, the usability of spatiotemporal SOC maps is currently very limited (Heuvelink et al. 2021; Meng et al. 2024; Venter et al. 2021). Following the review of Vaudour et al. (2022), the verifiability of predicted SOC changes therefore remains a critical issue, underlining the necessity to integrate soil data from long‐term monitoring programs.

Evaluating the results of local and regional soil monitoring, it has been shown that the drivers behind the cropland SOC dynamics are diverse (Wiesmeier et al. 2019). Recent spatiotemporal models have set a strong focus on land use changes as one important explanatory factor for SOC trends (De Rosa et al. 2024; Paul et al. 2020; Yang et al. 2023). As reported by several studies, however, SOC stocks have been generally decreasing in recent years, including the soils with continuous cropland management and no land use changes (Bellamy et al. 2005; Ciais et al. 2010; Heikkinen et al. 2013). To improve the knowledge about cropland SOC dynamics and provide unbiased and high‐resolution maps, SRC time series and variances in the spectral soil signal can be observed and directly linked to SOC changes. Some factors, like tillage or soil texture, can influence the cropland SOC as well as the soil reflectance and must be, therefore, analyzed in detail to improve the prediction of SOC trends (Castaldi et al. 2019; Dvorakova et al. 2020).

In summary, it remains challenging to predict and validate SOC trends based on EO data and repeated soil samples. In this study, we used data from Landsat and Sentinel‐2 satellite image time series to generate a datacube of SRC from 1986 to 2022 and trained a spatiotemporal SOC model based on soil samples from different long‐term monitoring sites. We aim to develop and assess EO‐based monitoring of cropland SOC trends by addressing the following research questions:

1. Is it possible to accurately predict short‐term SOC changes and long‐term SOC trends based on remote sensing data?
2. How is the uncertainty of SOC predictions based on SRC changing over time?
3. What factors influence the prediction and accuracy of cropland SOC trends?

## 2 Material and Methods

### 2.1 Research Area

**FIGURE 1:**
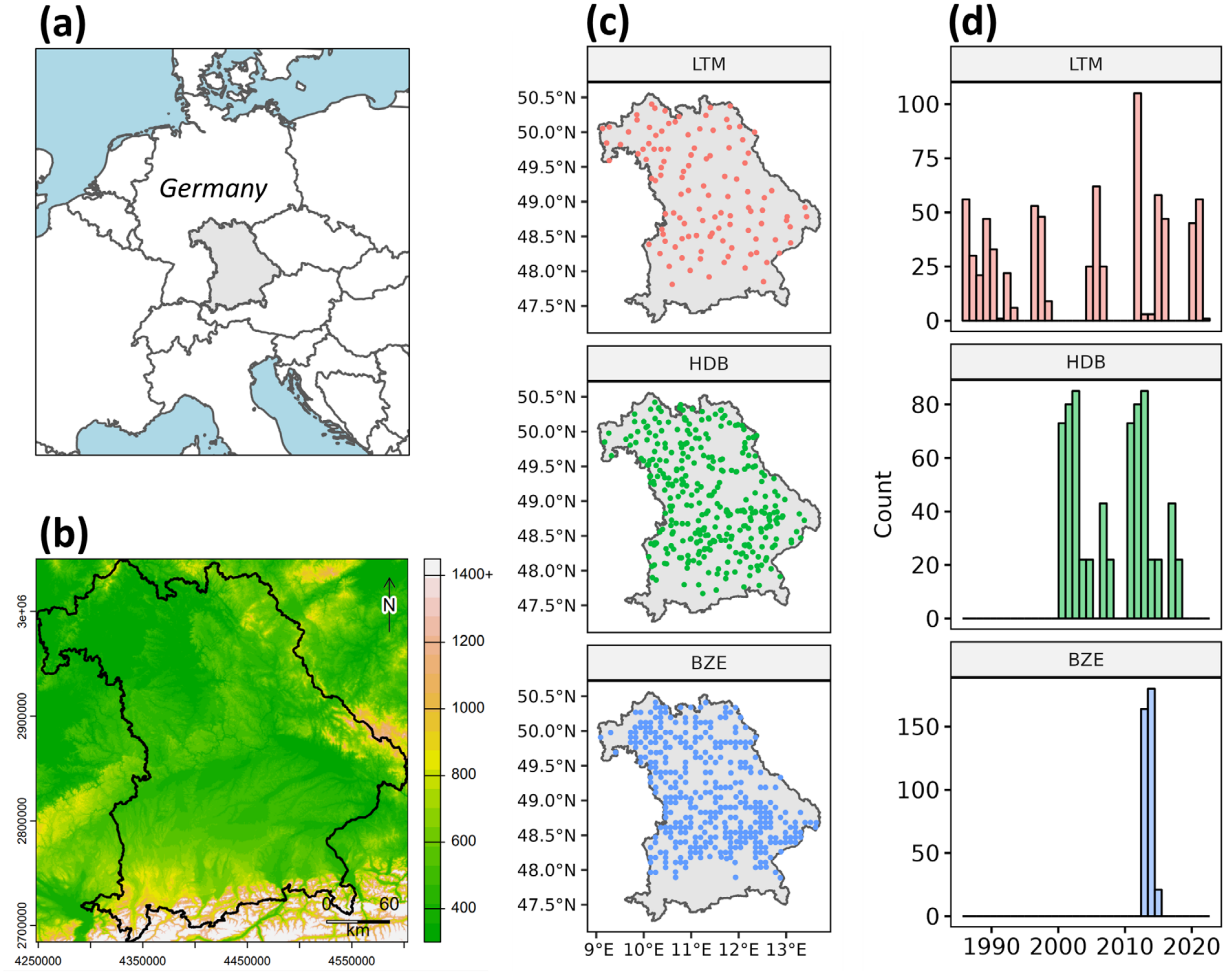
(a) Location of the research area in Central Europe. (b) Topographic map of Bavaria (m ASL). Spatial (c) and temporal (d) distributions of the SOC samples, used to train the model. LTM = Long‐term monitoring data (1986–2022); HDB = Monitoring data from the humus database (2001–2018); BZE = German Soil Inventory (2013–2015). Map lines delineate study areas and do not necessarily depict accepted national boundaries.

The research area of our study is located in the federal state of Bavaria, southeast Germany (Figure 1a). It is characterized by the Alps in the south, several low mountain ranges in the north, and east, and the lowland of the southern Molasse basin. The elevation ranges from 107 to 2962 m ASL and the climate is located in between maritime (northwest) and sub‐continental (southeast) influences (Figure 1b). In total, cropland accounts for about 34% of the land area. Grasslands and forests are predominantly located in mountainous regions with increasing altitude. Due to the diverse landscapes and parent materials, various soil classes are present in Bavaria. They include soils with well‐developed B horizons (Cambisols and Luvisols), as well as soils with initial soil formation (Leptosols). In some parts, high sand content led to the development of bleached A horizons (Podzols) while Vertisols formed on clay‐rich soils. Stagnosols and Gleysols are present in regions with shallow groundwater levels along valleys. In some regions of the area, the large streams originating in the Alps formed lowlands and induced the formation of natural bogs and fens (Histosols). Additional information about the soils in Bavaria can be found in Wiesmeier et al. (2013).

### 2.2 Soil Samples

For our study, we built on comprehensive soil samples from different programs (Table 1) (Broeg, Don, Wiesmeier, et al. 2024). An overview of the sampling locations and temporal distributions is given in Figure 1c,d. We included a total of 100 sites from a long‐term soil monitoring program (LTM) that were resampled seven times from 1986 to 2022 (Wiesmeier et al. 2024). The locations were selected to be representative of the climatic and geologic conditions in Bavaria (LfL 2022). They include 85 sites with continuous cropland management, seven sites with permanent crops, and eight sites with a change of cropland and grassland use within the observation period. The SOC content was analyzed in intervals of approx. 5 years: 1986–88, 1989–93, 1996–99, 2005–07, 2012–142,015–16, and 2020–22 (Figure 1d). For each site, 1000 m^2^ large parcels (~30 × 30 m) were laid out and marked with stationary magnets. The parcels were sampled at a depth of 0–15 cm, using four composite samples. A detailed description of the subsample layout is provided in LfL (2022). For laboratory analyses, the samples were dried (40°C), sieved (2 mm), and ground to 0.2 mm. The SOC contents (g kg^−1^) were calculated by analyzing the total soil C (Dumas method; Elemental analyzer) and subtracting the carbonate contents (Scheibler method). For our model, median SOC content values were calculated for each sampling date and linked to the central GPS coordinates of the plots.

**TABLE 1:** Descriptive statistics of the SOC samples (g kg^−1^), used for model training.

Soil data	*N* sites	*N* samples	Mean	Median	Min	Max	SD	IQR
ALL	796	1723	17.3	15.6	4.9	126.4	8.8	7.3
LTM	100	696	18.1	16.5	6.6	70.0	8.0	7.5
HDB	331	662	16.4	15.1	4.9	47.7	6.2	7.1
BZE	365	365	17.6	14.7	6.0	126.4	13.0	6.8

Abbreviations: IQR, interquartile range; *N* sites, number of cropland sites used in this study; *N* samples, number of training samples; SD, standard deviation.

Samples from an additional soil organic matter monitoring program (Humus Database = HDB) were used to complement the training data. This program includes a total of 331 cropland locations that were selected to represent the full spectrum of soil textures in Bavaria (Figure 1c). Each of the HDB sites was sampled twice, within exactly ten years. The first sampling campaign was conducted from 2001 to 2008 (Figure 1d). In each location, 7 m^2^ plots were marked with stationary magnets, and six composite samples were taken at a depth of 0–15 cm. SOC contents were determined with the same methods as described above.

Samples from the first German Soil Inventory (Bodenzustandserhebung Landwirtschaft = BZE) were added to further improve the spatial coverage of the model (Poeplau, Don, et al. 2020). They include a total of 365 cropland sites in Bavaria that were selected based on a regular 8 × 8 km grid (Figure 1c). The sites were sampled from 2013 to 2015 and have so far not been resampled. For each location, a 1 m profile was dug, and a distributed composite sample was collected at a depth of 0–10 cm. For laboratory analyses, the samples were oven‐dried (40°C) and sieved to 2 mm. The SOC content (g kg^−1^) was determined with dry combustion and by calculating the difference between total C and inorganic C contents (Poeplau, Jacobs, et al. 2020).

Furthermore, the LTM sites were grouped based on the mean SOC content (above/below 15 g kg^−1^; *n* = 64/36), tillage practice (tillage/reduced tillage; *n* = 56/44), and main soil texture (loam/sand/silt/clay; *n* = 47/30/13/10) to analyze the influence of different soil and management factors on the predicted and measured SOC trends. These factors were selected as they were (1) found to be connected to SOC trends in previous studies (Wiesmeier et al. 2024), (2) can significantly influence the soil reflectance signal in EO‐based SRC (Demattê et al. 2018), and (3) were available for all 100 LTM sites. The SOC threshold was selected according to Wiesmeier et al. (2013), who reported a median SOC content of 15 g kg^−1^ for cropland soils in Bavaria.

### 2.3 Measured SOC Trends and Changes

Based on the seven repeated samples from 1986/88 to 2020/22, linear regression analyses were conducted to derive the long‐term SOC trends for each of the 100 LTM sites. Using the sampling years (*x*) and the measured SOC contents (*y*), linear models were calculated to obtain the measured SOC trends (i.e., the slopes) in g kg^−1^ year^−1^. In addition, correlation coefficients (Pearson *r*) and the corresponding p‐values were derived from each of the measured linear regressions. Both values, SOC trends (g kg^−1^ year^−1^) and correlation coefficients (Pearson *r*), were later used to evaluate the temporal accuracy of the predictions (Section 2.6.2). For further analyses, the measured SOC trends were categorized into three groups based on the Pearson *r* and *p*‐values: increasing SOC trends: *r* > 0.5 & *p* < 0.1; decreasing SOC trends: *r* < −0.5 & *p* < 0.1; stable SOC trends: *r* > −0.5 & *r* < 0.5 & *p* > 0.1. In addition to the LTM long‐term trends, SOC changes between the two HDB sampling periods (2001/8 to 2011/18) were calculated for each site and used for additional validation of the temporal model accuracy.

### 2.4 Model Input

#### 2.4.1 Remote Sensing

**FIGURE 2:**
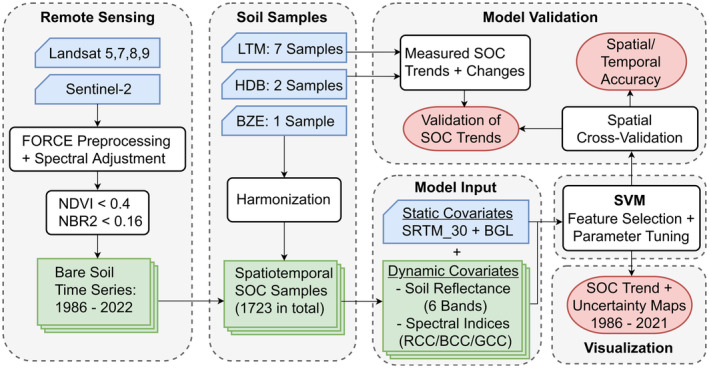
Schematic overview of the model workflow: input data is shown in blue; intermediate products are shown in green; results are shown in red.

Multispectral Landsat (5, 7, 8, 9) and Sentinel‐2 (A, B) images were combined to generate a time series of SRC for the observation period from 1986 to 2022. An overview of the workflow is shown in Figure 2. Data processing and radiometric corrections were conducted using the Framework for Operational Radiometric Correction for Environmental Monitoring (FORCE) (Frantz 2019). The FORCE Level‐2 module was used to create analysis‐ready bottom‐of‐atmosphere data. It includes atmospheric, topographic, as well as nadir BRDF adjustments that are described in detail in Frantz (2019). Clouds and shadows were removed using an optimized version of the FMASK algorithm (Frantz et al. 2018). We included all observations from 1986 to 2022 that were acquired between March to October and had cloud coverage below 70%. The images were converted to a common resolution of 30 m using nearest‐neighbor resampling. Six compatible spectral bands of the Landsat and Sentinel‐2 sensors were used for further analyses: Blue, Green, Red, Near‐Infrared (NIR), and Shortwave Infrared (SWIR1/SWIR2). To ensure that the spectral values are consistent over time and different sensors, the Landsat bands were linearly adjusted to the Sentinel‐2 sensor using the method described in Okujeni et al. (2024).

**FIGURE 3:**
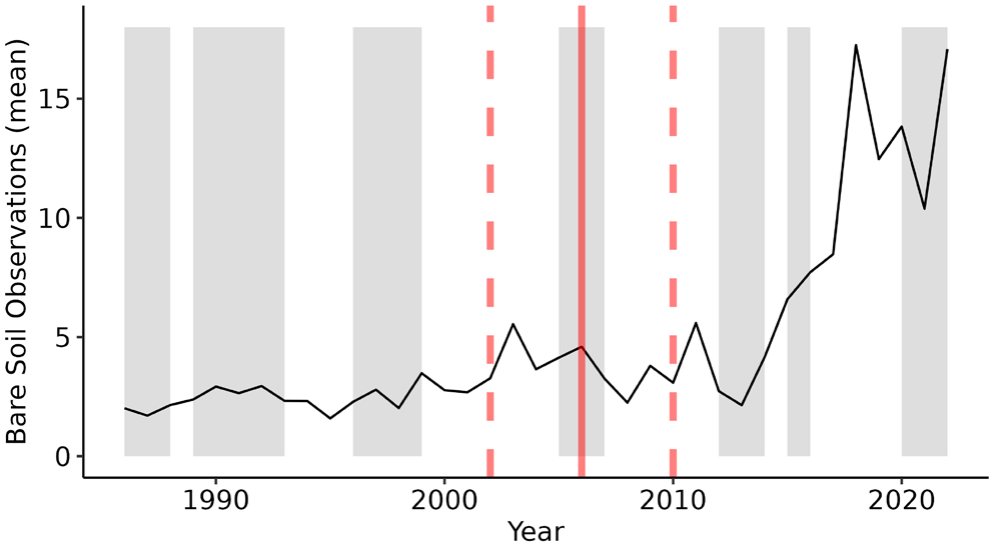
Mean number of bare soil observations (Landsat, Sentinel‐2) per year from 1986 to 2022 (black line), available at the SOC sampling locations. The seven LTM sampling periods are shown in gray. Red line = example of a single SOC measurement in time (2006); dashed lines = time window of ± 4 years, used to derive the SRC for the example sample.

In the next step, SRC were generated for each spatial and temporal location of the available soil samples (Figure 1) (Broeg, Don, Wiesmeier, et al. 2024). For this, the bare soil observations were filtered from the cloud‐free time series using the Normalized Difference Vegetation Index (NDVI) (Tucker 1979), the Normalized Burn Ratio 2 (NBR2) (Van Deventer et al. 1997), and the respective thresholds of 0.4 and 0.16 (Broeg, Don, Gocht, et al. 2024). We defined a time window of ± 4 years to derive an average SRC for each sampling date. This was done to increase the number of bare soil observations and to improve the quality of SRC, especially for earlier years of the observation period with less available satellite data (Vaudour et al. 2021; Zepp et al. 2023). Figure 3 illustrates the increasing availability of bare soil observations at the sampling locations as well as an example of the time window used to derive the SRC for one soil sample in time. Based on the selected observations, the mean soil reflectance was calculated for each band and used as input for the spatiotemporal model (Figure 2). The blue, green, and red chromatic coordinates (BCC, GCC, and RCC) were calculated to provide further information on relative soil color, in addition to absolute RGB values (Gillespie, Kahle, and Walker 1987).

#### 2.4.2 Static Covariates

Static covariates were added to the SOC model to provide spatial context and complement the dynamic SRC. We included a basic elevation model, provided by the Shuttle Radar Topography Mission with a resolution of 30 m (SRTM_30) (Farr and Kobrick 2000). Furthermore, we added the main soil‐landscape units in Bavaria (BGL) with 16 classes (1:5,000,000), as provided by the Federal Institute for Geosciences and Natural Resources (BGR 2008). The vector data was rasterized to match the common model resolution of 30 m and the categorical data was numerically encoded using an ordinal scale, provided by the BGR.

### 2.5 Spatiotemporal SOC Model

We combined the dynamic and static covariates with the available soil samples to train a spatiotemporal SOC model for the observation period from 1986 to 2022 (Broeg, Don, Wiesmeier, et al. 2024). As described in Section 2.4.1, we temporally matched bare soil observations and the dates of the SOC samples to generate an SRC data cube (Figure 2). Based on this, a single spatiotemporal Support Vector Machine (SVM) was trained using the SOC samples from all soil datasets and years (Cortes and Vapnik 1995). A forward feature selection was conducted to determine the best combination of covariates and reduce the risk of overfitting (Meyer et al. 2018). The following features were selected for the final model: RED, RCC, BCC, NBR2, SRTM_30, and BGL. The hyperparameter tuning was carried out using the hyperband method, as described by Li et al. (2017). The kernel of the SVM was set to “radial” and the following hyperparameter settings were selected for the model: Gamma = 0.055; Cost = 5.

### 2.6 Accuracy Assessment

The accuracy assessment of the SVM was conducted using a spatial 10‐fold cross‐validation to prevent repeated SOC samples from being used for model training and validation at the same time (Meyer et al. 2018). Based on the results, we calculated the Root Mean Square Error (RMSE), *R* ^2^‐values, and Lin's Concordance Correlation Coefficient (CCC) of the predictions. The Ratio of Performance to Deviation (RPD) was included to improve the comparability to other studies (Mcbratney and Minasny 2013).

#### 2.6.1 Temporal Model Accuracy

Repeated SOC samples from the LTM and HDB campaigns were used to analyze the consistency of the temporal model accuracy throughout the observation period. For this, the results of the spatial cross‐validation were grouped into the corresponding sampling periods to quantify and compare the temporal model accuracy (RMSE, *R* ^2^, CCC, and RPD). This includes seven SOC measurements for the LTM samples from 1986/88 to 2020/2022 and two measurements for the HDB samples from 2001/2008 to 2011/2018. In addition to the accuracy metrics, the prediction residuals of each sample period were compared to check if any temporal biases were present in the results. As the LTM only includes a limited number of sites with high SOC contents, the comparison of the temporal model accuracy was conducted twice, to reduce the influence of outliers on the results: including all samples and only considering samples with SOC contents below 40 g kg^−1^.

The signal‐to‐noise ratio was estimated based on the LTM time series to further analyze the relationship between model uncertainty and temporal SOC variability. For this, SOC changes between the first (1986/88) and the subsequent measurements (1989–2022) were quantified for each sampling year, and site. The standard deviation of the residuals was calculated to describe the temporal SOC change within the observation period. Next, the cross‐validated prediction residuals were quantified for each of the seven sampling periods. Based on the results, the standard deviation was calculated to derive spatial prediction uncertainty over time. Last, the signal‐to‐noise ratios were estimated for each sampling period by dividing the SOC change signal (i.e., standard deviation of the temporal residuals) and the prediction uncertainty (i.e., standard deviation of the prediction residuals). For further analyses, the calculation of the signal‐to‐noise ratio was repeated for the individual soil groups (mean SOC above/below 15 g kg^−1^), described in Section 2.2.

#### 2.6.2 Accuracy of the Predicted SOC Trends and Changes

The cross‐validated predictions were directly compared to the LTM measurements to evaluate the model's ability and accuracy to detect long‐term SOC trends and changes. For this, the predicted SOC trends (g kg^−1^ year^−1^) and Pearson correlation coefficients (r) were generated with the same methods described in Section 2.3. Calculations were performed for each pixel intersecting the LTM plots (Section 2.2), and the median values were taken to summarize the results for each site. The accuracy and significance of the predicted SOC trends and correlation coefficients were derived using the *R* ^2^ and corresponding p‐values, as well as an ANOVA and Tukey's HSD. Short‐term SOC changes were verified using repeated samples from the HDB. As all sites were resampled in the same ten‐year interval, measured and predicted SOC changes were directly compared to each other.

#### 2.6.3 Visualization of the Model

SOC contents in Bavaria were predicted from 1986 to 2021 to visualize the results of the spatiotemporal model (Broeg, Don, Wiesmeier, et al. 2024). For this, we generated an SRC every five years (1986, 1991, 1996, 2001, 2006, 2011, 2016, and 2021) and applied the spatiotemporal SOC model, described in Section 2.5 (Figure 2). In addition, uncertainty maps were generated by calculating the standard deviation of the ten SOC predictions, produced by the cross‐validation models (Section 2.6). Linear regressions (g kg^−1^ year^−1^) were calculated based on the SOC maps from all years to derive SOC trends for each cropland pixel in the research area.

## 3 Results

### 3.1 Overall Model Accuracy

**FIGURE 4:**
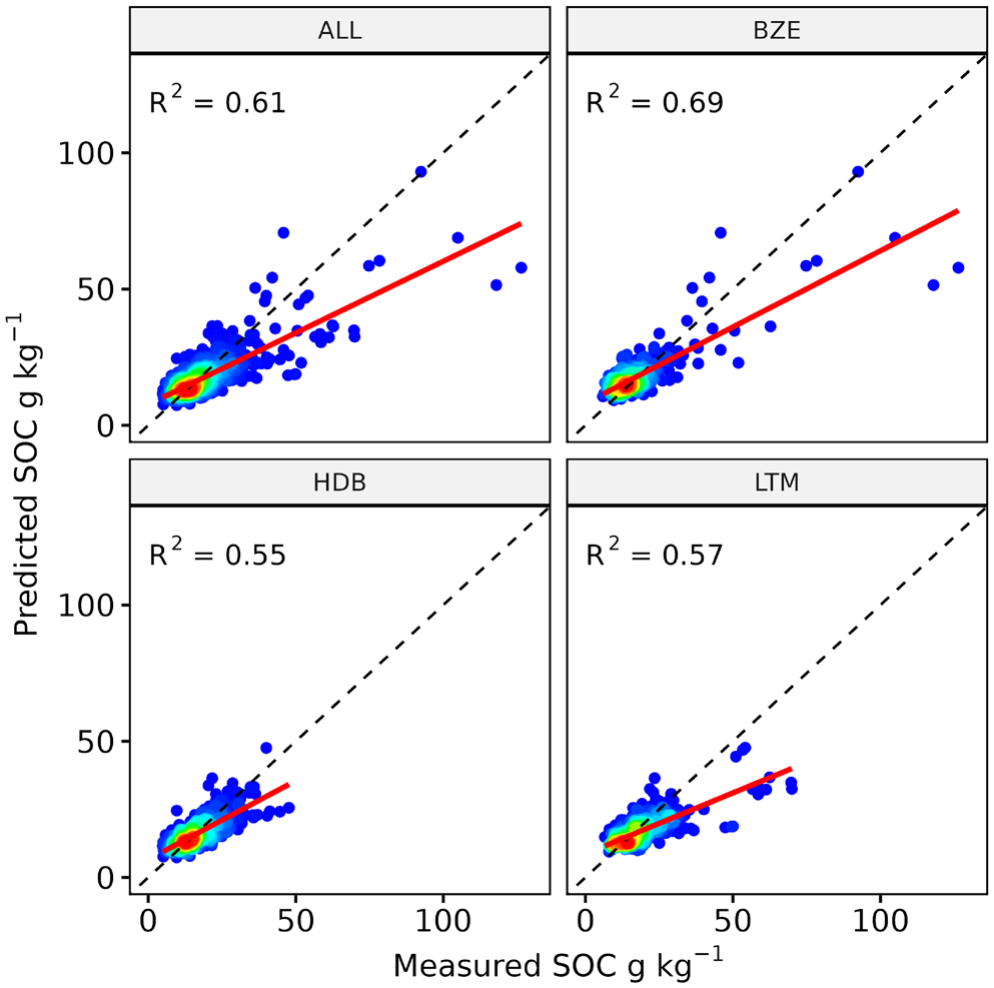
Cross‐validated predictions of the three spatiotemporal SOC datasets (LTM, BZE, HDB), and the combined samples (ALL). Regression lines are shown in red; 1:1‐line is shown in black (dashed).

Based on the results of the spatial cross‐validation, the developed spatiotemporal SOC model produced an overall *R* ^2^ value of 0.61 and an RMSE of 5.6 g kg^−1^ (Table 2, Figure 4). Looking into the different soil datasets individually, the results ranged between 0.55 and 0.69 (*R* ^2^), and 4.2 and 7.5 g kg^−1^ (RMSE). According to the CCC (0.77) and RPD (1.73), the BZE data produced the highest prediction accuracy. The repeated soil samples from the LTM and HDB showed similar results with *R* ^2^ values of 0.55 and 0.57 and RPD values of 1.44 and 1.49. In general, the HDB samples had the lowest SOC range (Table 1) and showed the best RMSE (4.2 g kg^−1^). As illustrated by the high CCC value (0.72), the predictions were closer to the 1:1 line, compared to the LTM samples (0.65). The regression lines show a general tendency for underpredictions, especially for high SOC contents (Figure 4).

**TABLE 2:** Cross‐validation accuracy of the three spatiotemporal SOC datasets (LTM, BZE, HDB) and the combined samples (ALL).

Soil data	*N* samples	*R* ^2^	CCC	RMSE	RPD
ALL	1723	0.61	0.72	5.58	1.57
LTM	696	0.57	0.65	5.56	1.44
HDB	662	0.55	0.72	4.15	1.49
BZE	365	0.69	0.77	7.54	1.73

### 3.2 Temporal Changes in Model Accuracy

**FIGURE 5:**
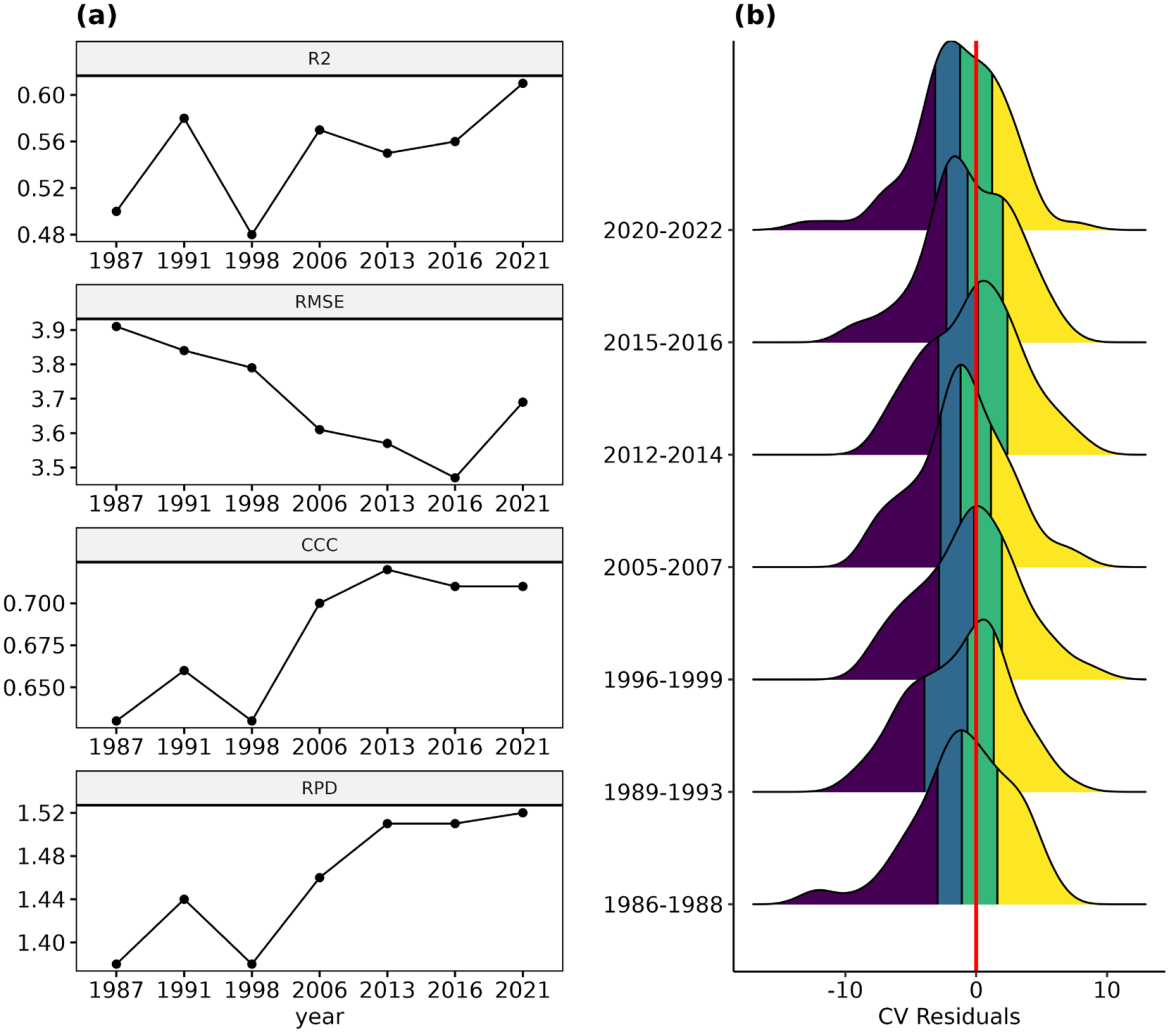
(a) Temporal trends of the model accuracy (*R* ^2^, RMSE, CCC, and RPD), based on the repeated LTM samples below 40 g kg^−1^. (b) Distributions of the prediction intervals; quartiles are illustrated with colors.

Based on the seven repeated LTM samples from 1986/88 to 2020/22, the SOC model exhibits a high temporal consistency for the samples below 40 g kg^−1^ (Figure 5). The *R* ^2^ shows slight fluctuations over time and ranges between values of 0.48 (1996/99) and 0.61 (2020/22) (Figure 5a). According to the RMSE, the prediction accuracy incrementally improved by around 8% throughout the observation period (~ 3.9 to 3.6 g kg^−1^). Similar tendencies are visible for the CCC and RPD, especially for later years after 2006. In comparison, the model produced slightly better results when considering all samples, with *R* ^2^ values between 0.57 (2005/07) and 0.67 (2015/16) (Figure S1). While the CCC and RPD showed similar increasing tendencies, the RMSE mostly remained stable with values around 5 g kg^−1^. Based on the samples below 40 g kg^−1^, the prediction residuals are normally distributed and reveal a high temporal consistency (Figure 5b). For most years, a slight tendency for underpredictions is visible in the data. Scatterplots, with the results from each sampling period, are shown in Figure S2. Comparing the results of the two HDB sampling periods from 2001 to 2008, and from 2011 to 2018, only slight temporal variations are visible (Figure S3). The *R* ^2^ values remain mostly constant (0.56/0.55), while the RMSE decreased slightly, from 4.3 to 3.9 g kg^−1^.

### 3.3 Signal‐to‐Noise Ratio

**FIGURE 6:**
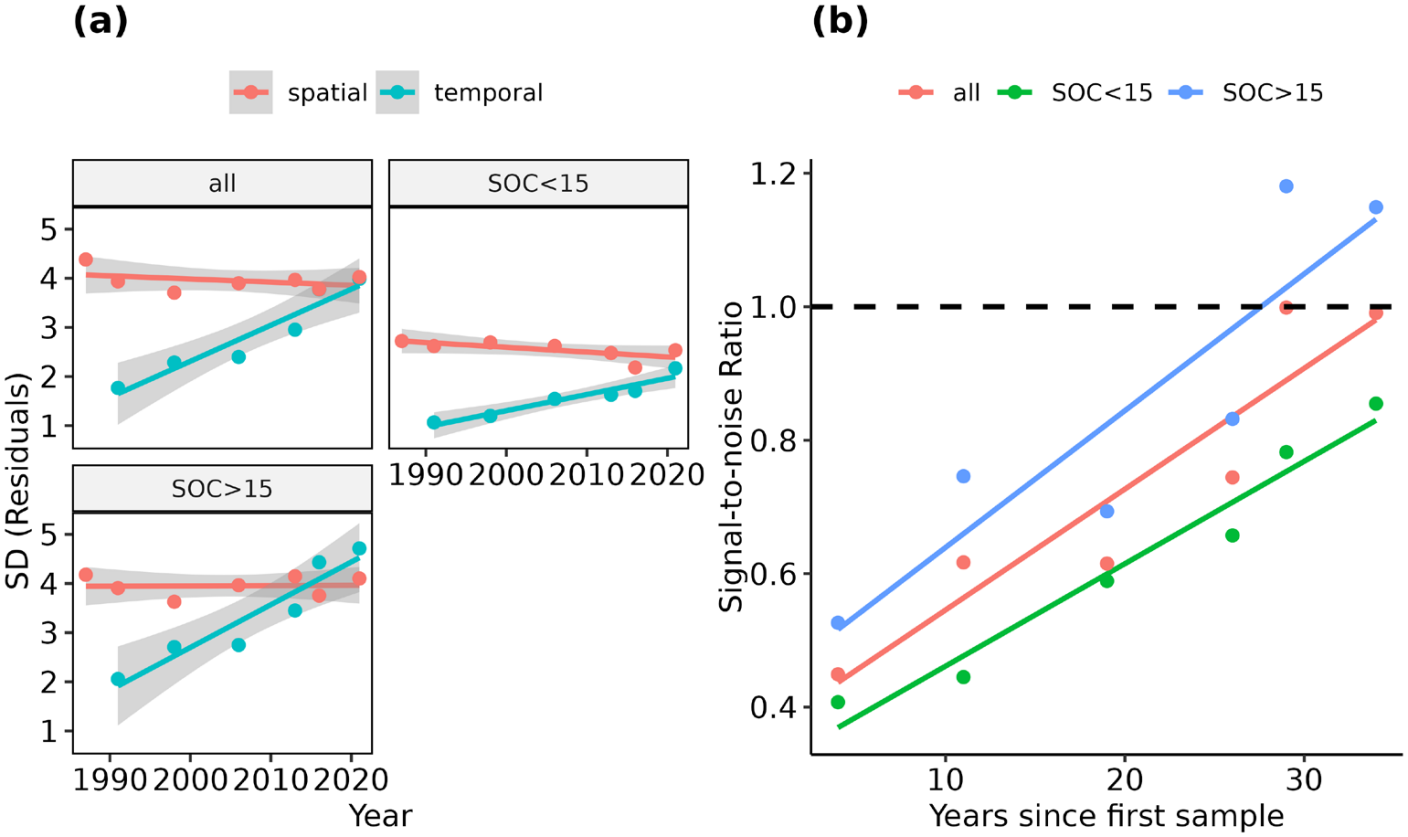
Standard deviation (SD) of the spatial (prediction uncertainty) and temporal (SOC changes) residuals of the LTM samples (a). The plots were grouped according to the mean SOC contents of the sites: above/below 15 g kg^−1^. Signal‐to‐noise ratio throughout the observation time, as derived from the temporal and spatial SOC residuals (b).

Overall, the measured SOC change steadily increased since the initial SOC samples were taken in 1986/88 (Figure 6a). This is illustrated by the standard deviation of the temporal residuals, which doubled from around 2 (1990) to 4 g kg^−1^ in 2020. In contrast, the standard deviation of the spatial prediction residuals (i.e., the prediction uncertainty) mostly remained stable throughout the observation period with values around 4 g kg^−1^. As a result, the signal‐to‐noise ratio incrementally improved over time and reached a value of around 1 in 2016, roughly 30 years after the initial SOC samples were collected (Figure 6b).

For further analysis, the calculation of the signal‐to‐noise ratio was repeated for the LTM sites with mean SOC contents above and below 15 g kg^−1^ (Section 2.2). In both cases, the results had similar but slightly shifted trends (Figure 6a). Sites with mean SOC contents above 15 g kg^−1^ showed larger SOC changes over time (temporal residuals) and generally produced higher signal‐to‐noise ratios. The opposite is true for the remaining sites with SOC values under 15 g kg^−1^. Although the spatial residuals were much lower, the temporal SOC changes were too small and resulted in ratios under 1, even for long time series of 35+ years (Figure 6b).

### 3.4 Prediction of SOC Trends and Changes

**FIGURE 7:**
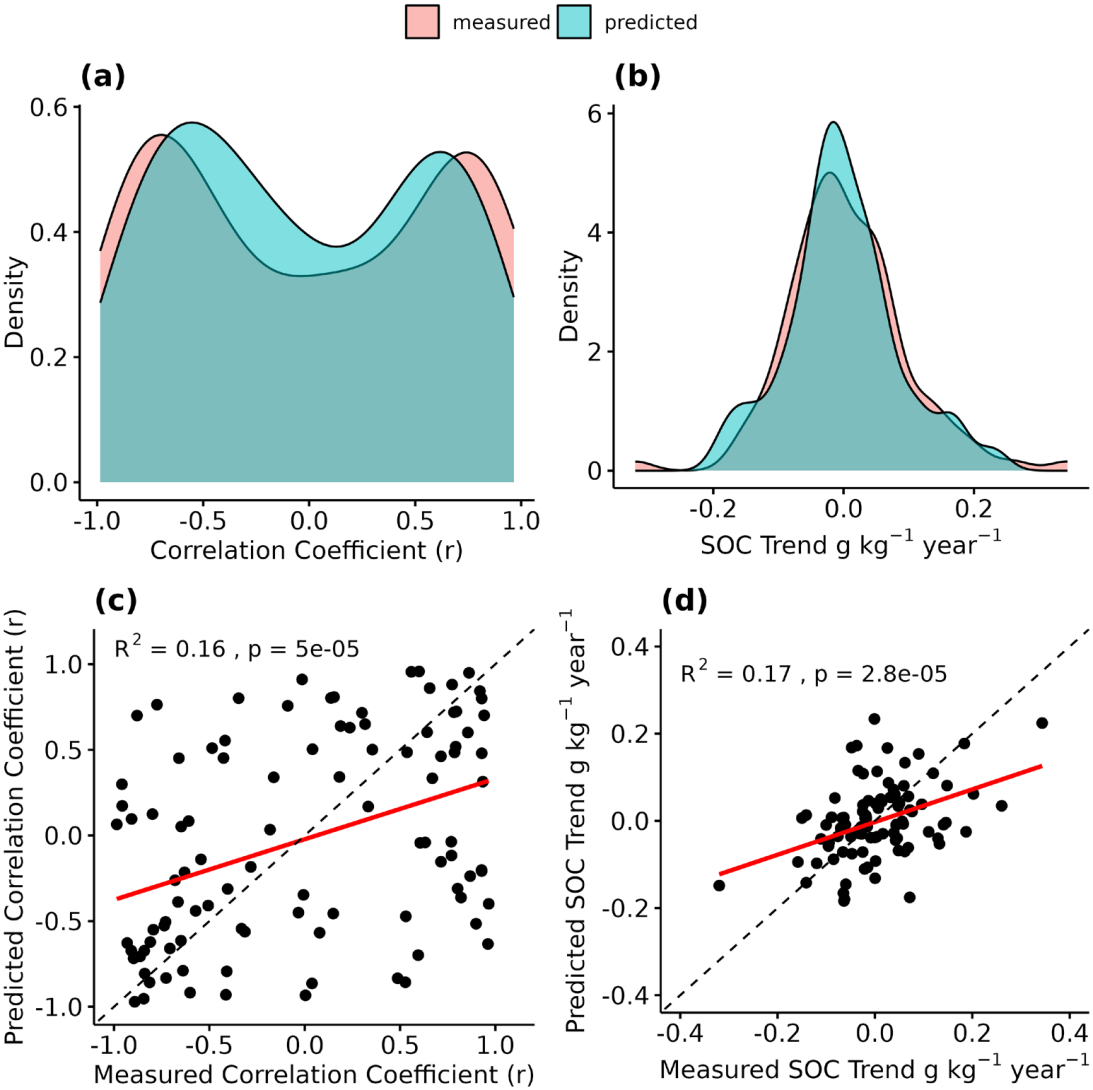
Distribution of the measured and predicted correlation coefficients (Pearson *r*) (a) and SOC trends (g kg^−1^ year^−1^) (b). Direct comparison of the results (c + d). Regression lines are shown in red; 1:1‐lines are shown in black (dashed).

Out of the 100 observed LTM sites, exactly 50 showed increasing and decreasing SOC trends (Figure 7a,b). In around 70% of all sites, the Pearson *r* values were below −0.5 or above 0.5. A similar distribution was produced by our model. Here, 47 sites showed positive trends while the r values were below −0.5 or above 0.5 in around 60% of the cases. The direct validation, however, only revealed a low (*R* ^2^ = 0.16) but significant (*p* < 0.0001) accuracy (Figure 7c). The measured SOC trends showed a normal distribution and median values close to 0 g kg^−1^ year^−1^ (Figure 7b). For most LTM sites, the values were in the range between −0.2 and 0.2 g kg^−1^ year^−1^, with few outliers in both directions. In general, the predicted values showed a similar distribution and range, with a median value slightly below 0 g kg^−1^ year^−1^. Similar to the r values, a direct comparison of the trends only yielded a low *R* ^2^ value of 0.17 (Figure 7d).

**FIGURE 8:**
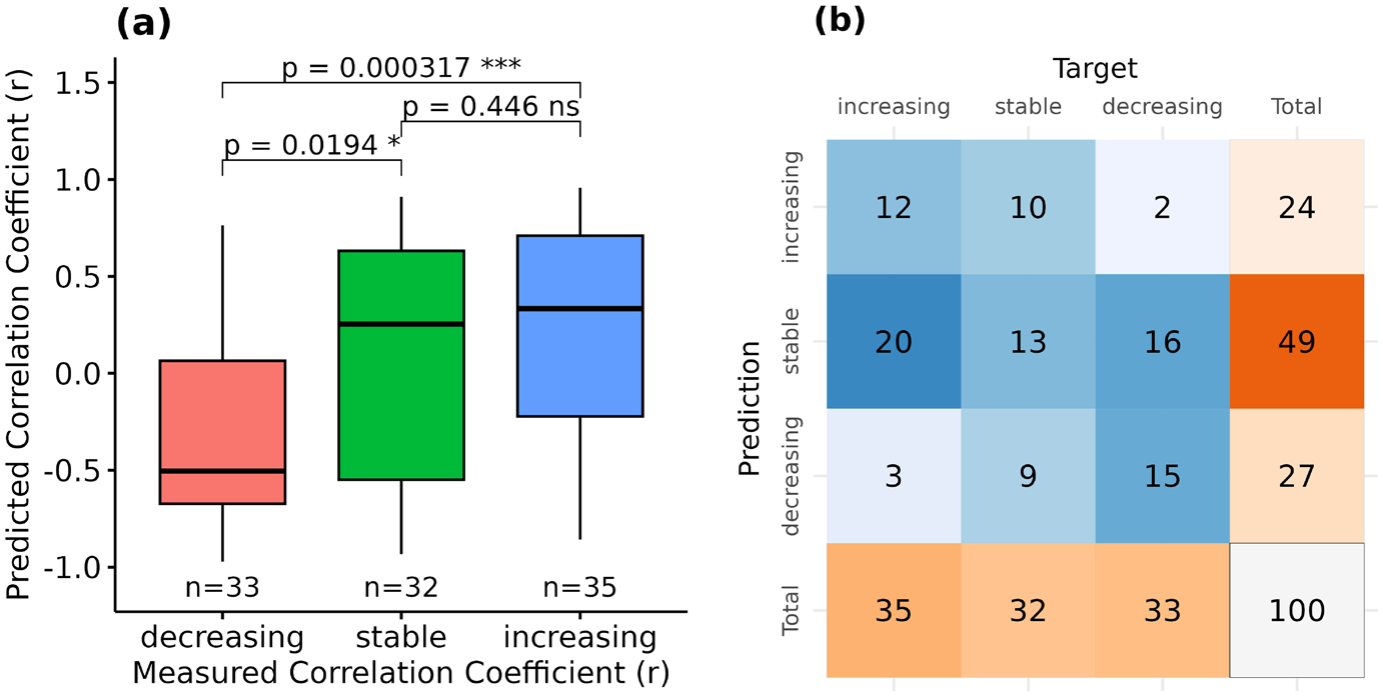
Boxplots of the measured and predicted correlation coefficients (Pearson *r*) (a). Decreasing: *R* < −0.5; stable: *R* > −0.5 and < 0.5; increasing: *r* > 0.5. Confusion matrix of the predicted and measured correlation coefficients (Pearson *r*) (b).

Measured and predicted SOC trends were classified into three groups to further analyze the results: decreasing, stable, and increasing (Section 2.3). In general, it was possible to differentiate between increasing and decreasing trends with high significance (*p* < 0.001) (Figure 8a). While significant results were also visible between the stable and decreasing classes (*p* < 0.05), no differences were found between the stable and increasing classes. As illustrated by the confusion matrix in Figure 8b, misclassifications between the decreasing or increasing classes occurred in only 5% of the observed sites.

The results of the short‐term SOC changes, based on the HDB plots, are shown in Figure S4. Within the 10‐year intervals, most sites showed SOC changes between −4 and 4 g kg^−1^. The measured values were normally distributed and showed a median value close to 0 g kg^−1^. Although the model generally produced a similar distribution and range, no significant correlation between the measured and predicted SOC changes was found.

### 3.5 Influence of Additional Factors on Long‐Term SOC Trends

**FIGURE 9:**
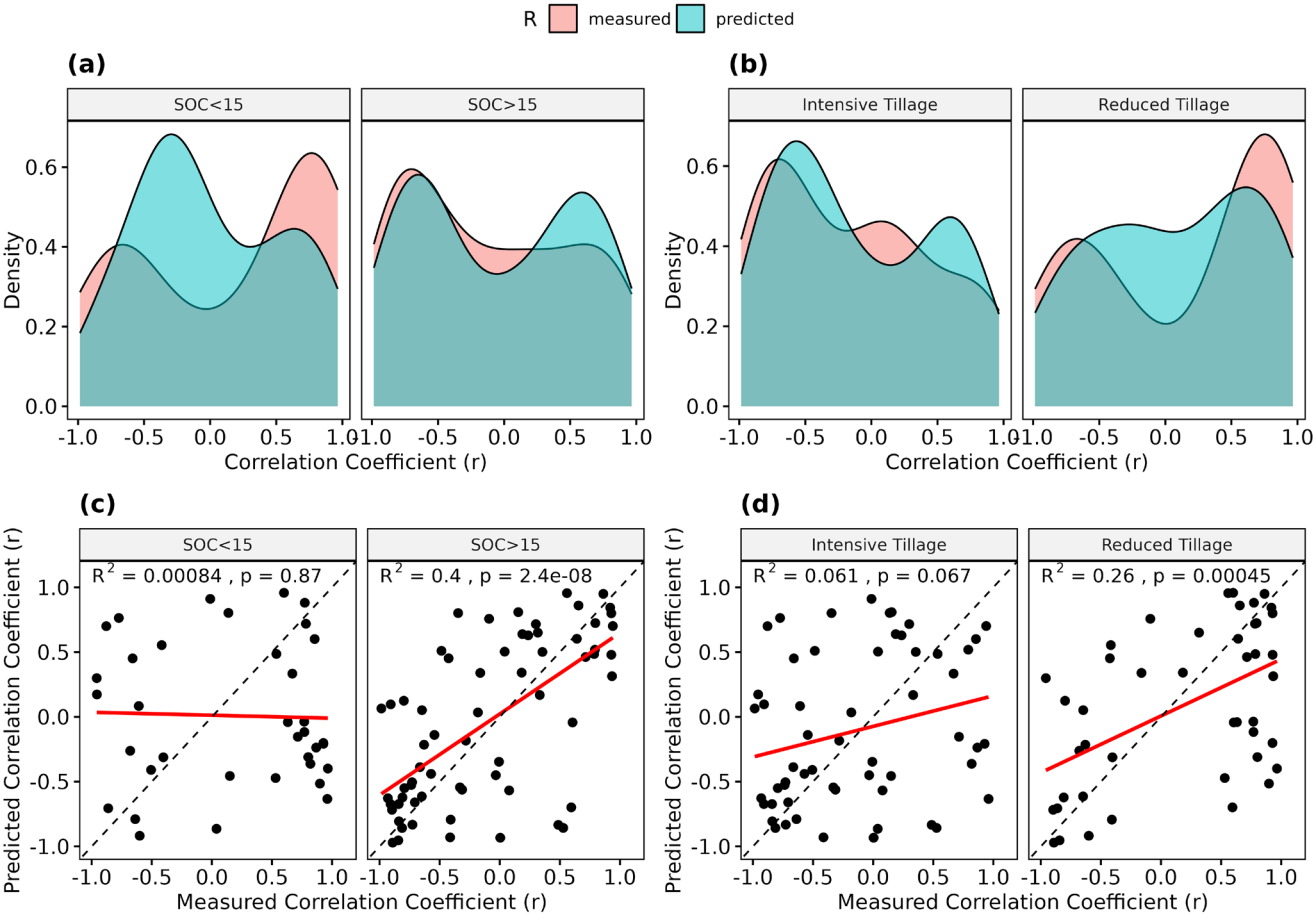
Distribution of the measured and predicted correlation coefficients (Pearson *r*), grouped by mean SOC content (above/below 15 g kg^−1^) and tillage regime (intensive/reduced) (a + b). Corresponding scatterplots of the results (c + d). Regression lines are shown in red; 1:1‐lines are shown in black (dashed).

**FIGURE 10:**
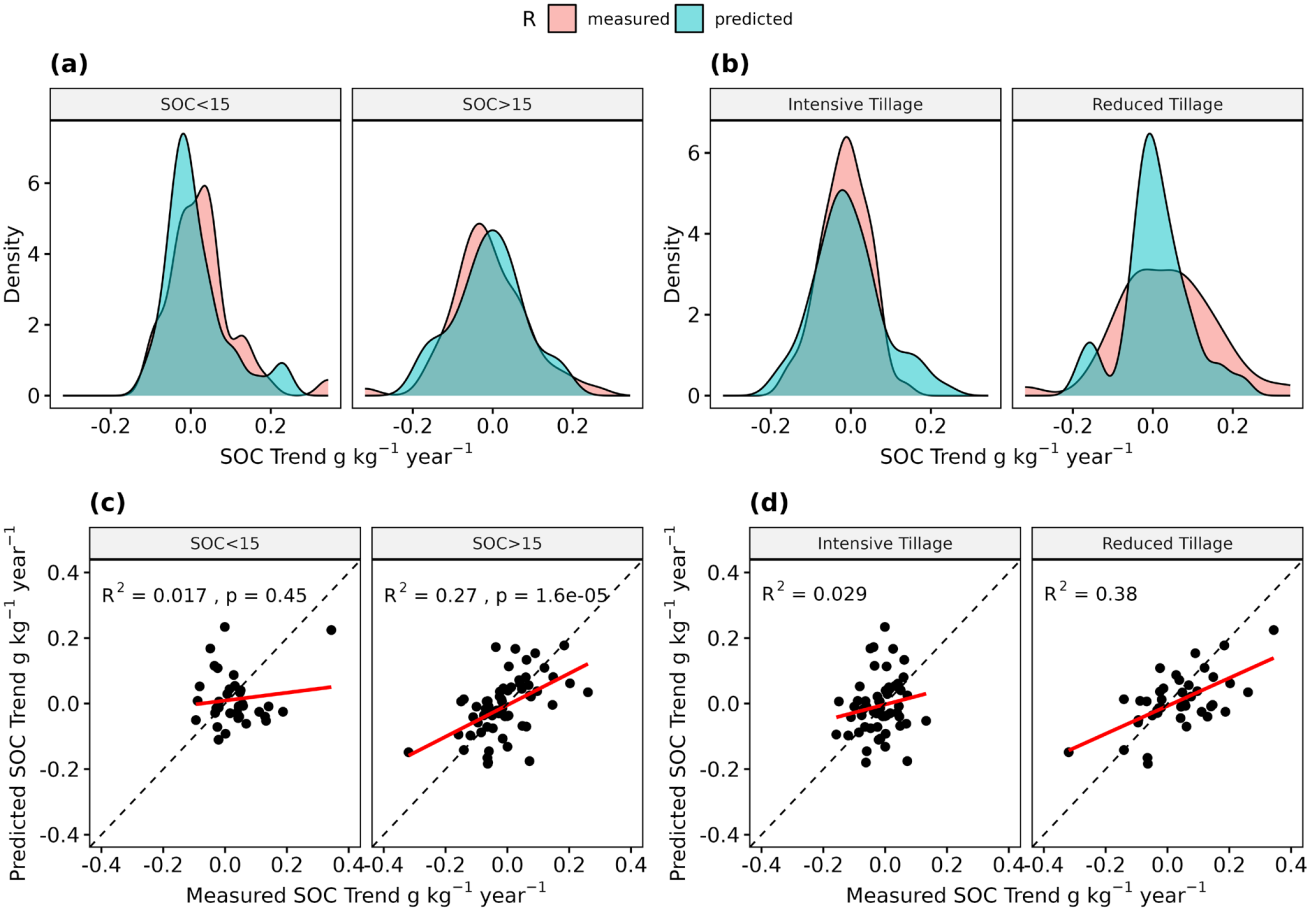
Distribution of the measured and predicted SOC trends (g kg^−1^ year^−1^), grouped by mean SOC content (above/below 15 g kg^−1^) and tillage regime (intensive/reduced) (a + b). Corresponding scatterplots of the results (c + d). Regression lines are shown in red; 1:1‐lines are shown in black (dashed).

The LTM sites were categorized based on their mean SOC content, tillage intensity, and soil texture to further analyze the results of the long‐term SOC trends (Section 2.2). In general, the sites with a mean SOC content above 15 g kg^−1^ showed lower correlation coefficients (Figure 9a), and a broader distribution of SOC trends (Figure 10a). In comparison, sites with SOC contents below 15 g kg^−1^ often showed SOC trends close to 0 g kg^−1^, indicating no or little SOC changes (Figure 10a). In general, the model produced much better results for the LTM sites with mean SOC contents above 15 g kg^−1^ (*R* ^2^ = 0.27/0.4), while the remaining sites (SOC < 15 g kg^−1^) showed no significant results (Figures 9c and 10c).

Overall, the presence of reduced tillage resulted in a higher number of positive SOC trends (Figure 9b). This was the case for both, the predicted and measured values. In comparison to the sites with intensive tillage, the trends showed a broader value distribution and higher absolute SOC changes (Figure 10b). In general, the spatiotemporal model produced much higher accuracies for the sites with reduced tillage, with *R* ^2^ values of 0.26 and 0.38 (Figures 9d and 10d).

Compared to the other factors, the influence of the main soil texture on the results is less clear (Figure S5). Loam and sand soils, which include most of the LTM sites, show similar results to the overall accuracy (*R* ^2^ = 0.12/0.16). In contrast, LMT sites with clay soils produced a significantly higher (*R* ^2^ = 0.6), while silt soils produced a lower accuracy (*R* ^2^ = 0.09). Both of these classes are, however, most likely influenced by the low number of available sites (*n* = 10/13).

### 3.6 SOC Predictions and Trend Map

**FIGURE 11:**
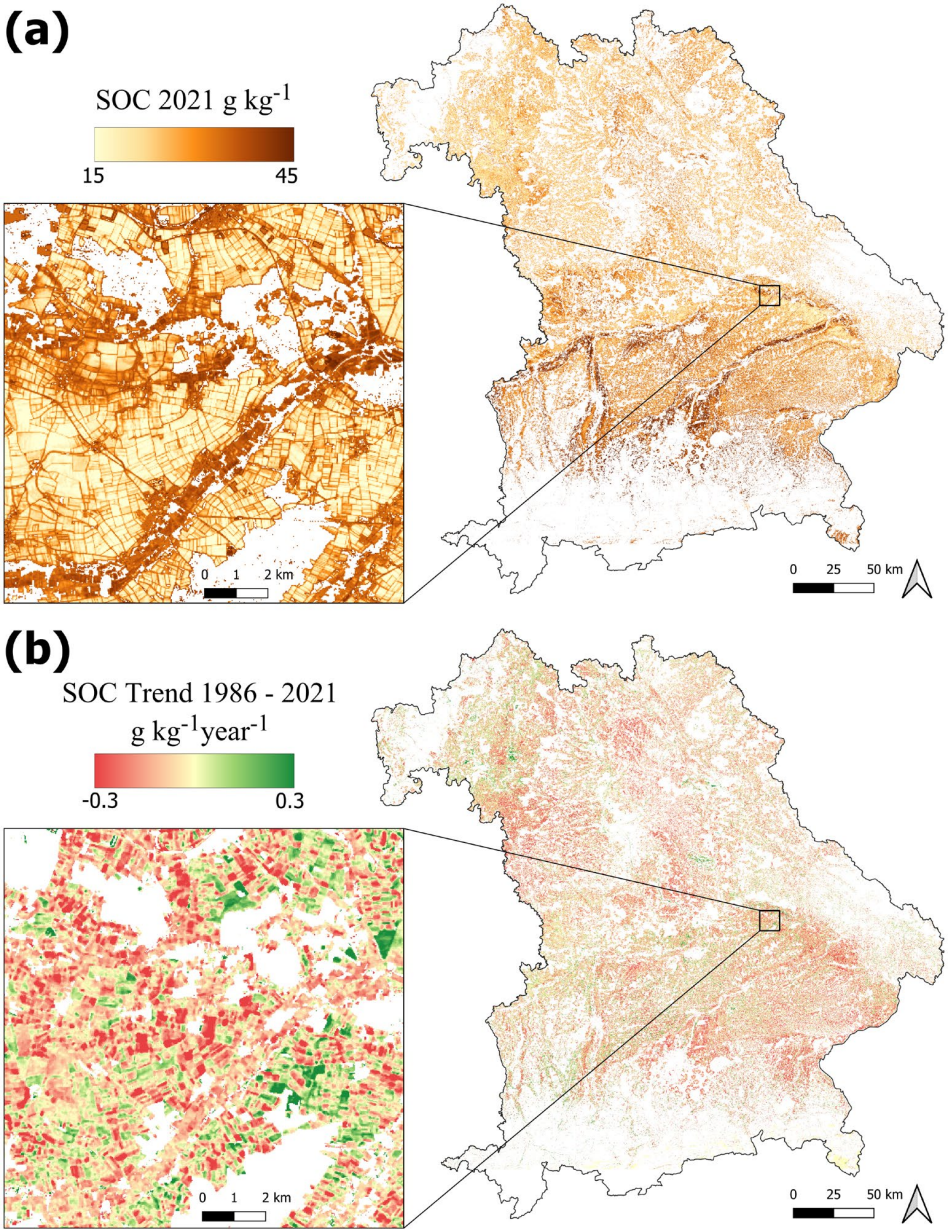
(a) Map of the predicted cropland SOC content in 2021 (g kg^−1^). (b) SOC trend map (g kg^−1^ year^−1^), calculated based on the individual SOC predictions from 1986 to 2021. Map lines delineate study areas and do not necessarily depict accepted national boundaries.

The spatiotemporal model was used to predict the SOC content in Bavaria every five years from 1986 to 2021. Looking at the most recent SOC map in 2021, the highest SOC contents are present in the south and connected to the large stream, originating in the Alps (Figure 11a). A similar spatial distribution is visible in the corresponding uncertainty map (Figure S6). An example of the differences, visible between the individual SOC maps, is provided in Figure S7. Based on the individual predictions, SOC trends were calculated for the full observation period from 1986 to 2021 (Figure 11b). While increasing trends are present across all regions, the resulting map shows that the SOC of most cropland soils in Bavaria is stable or slightly decreasing. Regional patterns as well as small‐scale differences are visible throughout the research area. As illustrated in Figure 11b, contrasting SOC trends were predicted at a field scale, even for neighboring cropland sites with similar SOC contents.

## 4 Discussion

### 4.1 Overall Model Accuracy

We combined repeated soil samples from 1986 to 2022 and EO data to train a spatiotemporal SOC model for Bavaria. The overall *R* ^2^ value of 0.61 is comparable to static large‐scale SOC maps in Germany (Broeg, Don, Gocht, et al. 2024), and Bavaria (Zepp et al. 2021), illustrating that it is possible to construct spatiotemporal SOC models without compromising prediction accuracy (Chen et al. 2022). Overall, the three soil data sets yielded varying results (Figure 4, Table 2). Comparing the *R* ^2^ values of the HDB (0.57) and BZE (0.69), it is clear that the higher range and standard deviation of the BZE samples (Table 1) significantly influenced the results. While BZE was sampled on a regular grid (Figure 1c) and included more SOC values above 40 g kg^−1^, the HDB sites were selected to represent the soil textures in Bavaria. In both cases, the median SOC values are similar (Table 2), and the CCC values (0.77/0.72) support the conclusion that the overall accuracy was high (Table 2). Although the LTM includes a lower number of sites, they express a high spatial representativeness and are evenly distributed across the research area (Figure 1c). Considering that the samples were collected within a time frame of almost four decades, including years with limited EO data (Figure 3), the model produced a good but slightly lower overall accuracy (CCC = 0.65 and RPD = 1.44). Similar to previous studies, we found a limited accuracy and tendency for underpredictions at high SOC contents (Figure 4) (Feeney et al. 2022; Zepp et al. 2021). Considering the low availability of SOC samples above 40 g kg^−1^, it can be concluded that additional soil data is needed to improve the prediction accuracy for high SOC contents. Further tests are necessary to evaluate if EO data provides sufficient information on the full range of SOC values, including organic soils, or needs to be extended by other covariates (Feeney et al. 2022).

### 4.2 Temporal Changes in Model Uncertainty

Quantifying the temporal model uncertainty is an important prerequisite in understanding and improving the prediction of SOC trends. As pointed out by Croft, Kuhn, and Anderson (2012), variances between different satellite sensors represent a challenge to the transferability and accuracy of spatiotemporal SOC models. Accordingly, spectral adjustments were conducted in this study and several other works and used to preprocess the remote sensing data for the SOC models (Meng et al. 2024; Venter et al. 2021). Without a validation based on repeated SOC samples, however, it is difficult to provide unbiased information about the consistency of the temporal model accuracy. Our results show that the model accuracy and residual distribution are comparable for all seven sampling periods from 1986 to 2022 (Figure 5). This is an important prerequisite, as a temporal bias could result in artifacts when deriving the SOC trends based on the model predictions.

With an RPD of 1.44, the validation of the LTM samples revealed a slightly lower accuracy compared to the other soil data sets (Table 2). Taking into account the temporal model accuracy of the LTM samples, different explanations for this can be derived: First, improvements of the RMSE and CCC over time (Figure 5) seem to coincide with the strongly increasing number of bare soil observations (Figure 3). This is especially true for the years after 2015, following the launch of the Sentinel‐2 satellites, and could have positively affected the quality of the SRC. For the more recent sampling periods, the RPD of the LTM samples increased to values above 1.5 which are comparable to the other soil data sets (Figure 5, Figure S1). Second, slight adjustments in the sampling design of the LTM plots could have led to better results in later years. This is supported by the fact that the coefficient of variation for the subsamples has been decreasing throughout the years (LfL 2022). In summary, our results underline that the generation of SRC based on a moving window is suitable to increase the temporal model consistency, especially for years with varying availability of EO data. The slightly lower accuracy in the early years could have, however, negatively affected the detection of long‐term SOC trends. Further research is necessary to test if the accuracy can be improved without sacrificing on the temporal model resolution.

### 4.3 Signal‐to‐Noise Ratio

Using the repeated LTM samples, we were able to show that the signal‐to‐noise ratio of measured SOC changes and model uncertainty significantly increased throughout the observation period (Figure 6b). These findings highlight the large potential of our approach to monitoring SOC trends but also underline the necessity to analyze long observation periods. Based on our estimates, a minimum number of 25 years was necessary to obtain signal‐to‐noise ratios with values above 1. Considering that SOC changes in cropland soils are generally very slow (Ciais et al. 2010), these findings support the conclusion that it is easier to predict long‐term SOC trends rather than short‐term changes. Based on experiments, Smith (2004) demonstrated that in soils with low carbon inputs, up to 15 years are necessary to measure significant changes. This could be one reason why we were unable to verify the SOC changes in the HDB samples that were repeated after 10 years (Figure S4). Furthermore, we were also able to demonstrate a connection between the mean SOC contents and the ability to predict SOC trends. In soils with contents above 15 g kg^−1^, the measured SOC changes outweighed the higher prediction uncertainty and increased the signal‐to‐noise ratio (Figure 6). In contrast, soils with lower SOC contents also produced a lower signal‐to‐noise ratio, increasing the necessary time frame and prediction accuracy to detect changes. Summarizing the results, we were able to demonstrate three key factors that influence the ability to predict SOC trends and should be reported when applying spatiotemporal SOC models: (1) the mean SOC contents and absolute SOC changes; (2) the temporal model uncertainty; and (3) the length of the observation period.

### 4.4 Prediction and Validation of SOC Trends and Changes

There is an increasing demand for EO data and spatially explicit information on SOC trends with existing and upcoming carbon farming schemes and their role in contributing to carbon removal and climate change mitigation (Amelung et al. 2020; Lehmann et al. 2020; Paustian et al. 2019). To increase the usability of EO‐based models, it is necessary to validate the predictions with measured SOC trends and changes. Due to the low availability of large‐scale soil inventories with high‐quality repeated soil samples, however, recent studies rely on indirect measures to validate the accuracy of the predicted SOC changes (Heuvelink et al. 2021; Meng et al. 2024; Venter et al. 2021). Our findings highlight that the general model uncertainty is insufficient to verify spatiotemporal SOC models and could lead to an over‐optimistic interpretation of the results. Although the cross‐validation produced good results in all cases (*R* ^2^ = 0.61), the direct validation of the predicted long‐term SOC trends revealed a much lower accuracy (*R* ^2^ = 0.16/0.17) (Figure 7). This is the case even though the predicted and measured SOC trends share a nearly identical distribution of values.

Despite the high uncertainty, our results underline that EO data is a valuable asset for deriving long‐term trends in cropland soils. As shown in Figure 8b, the model rarely predicted trends opposite to the measurements, illustrating that a clear SOC signal is present in the data. In general, the predicted r values showed significant differences for sites with increasing and decreasing SOC trends (Figure 8a). Using the validated spatiotemporal model, it is possible to generate high‐resolution information on the cropland SOC dynamics from the year 1986 onwards. As illustrated in Figure 11b, SOC trends can be derived and analyzed at a field scale. Even though the results show that the signal is not strong enough to detect SOC changes in all cases, they significantly enhance the data density and understanding of the cropland soil dynamics within the observed timeframe. Thus, for the first time, it will be possible to estimate long‐term SOC trends for large regions in very high resolution.

### 4.5 Influence of Additional Factors on Long‐Term SOC Trends

Considering the high uncertainty associated with the predicted SOC trends, it is necessary to further analyze and understand the contributing factors. In general, the mean SOC contents of the sites influenced the SOC trends as well as the accuracy of the predictions. As illustrated in Figure 9, the observed LTM sites with low SOC contents predominantly showed increasing trends, while the opposite is true for sites with contents above 15 g kg^−1^. Similar results have been reported in other studies and can be partly linked to historic land use changes that have influenced SOC dynamics and trends for decades (Bellamy et al. 2005; Heikkinen et al. 2013; Lettens et al. 2005). Furthermore, sites with SOC contents above 15 g kg^−1^ showed higher accuracy in the predicted trends (*R* ^2^ = 0.4), while no significant results were found for the remaining sites (Figure 9). Similarly to the signal‐to‐noise ratio (Figure 6), absolute SOC changes may be too small to be detectable by the remote sensing data, resulting in low accuracy. This is supported by the distributions of the predicted and measured values in Figure 10 and the fact that SOC trends were often close to 0 g kg^−1^ year^−1^. In general, our findings show that the model is more likely to detect the changes in soils with larger C‐stocks, which are also at higher risk of SOC loss (Bellamy et al. 2005).

Additionally, we analyzed the influence of tillage practices on the predicted and measured SOC trends. Several studies reported increasing SOC contents on surface soil layers, following the implementation of reduced tillage measures on cropland soils (Bai et al. 2019; Haddaway et al. 2017; Ogle et al. 2019). It is often argued that this is due to the accumulation of the SOC in the uppermost centimeters of the topsoil, while the SOC content below is decreasing with little effect on the overall SOC stocks of the total soil profile (Baker et al. 2007; Meurer et al. 2018). As the SOC samples were taken at a depth of 0–15 cm, sites with reduced tillage showed increasing SOC trends in most cases (Figure 9). Most of these SOC trends were correctly predicted as increasing, and the accuracy of the predictions is significantly higher, compared to the sites with conventional tillage (Figure 9). This result is surprising since reduced tillage can introduce organic soil cover (litter, crop residuals, etc.) and influence soil reflectance beyond the SOC change signal (Dvorakova et al. 2022). It is possible that these changes in the surface conditions lead to higher SOC predictions and generally increase the chances of predicting positive trends. Our findings underline the importance of tillage on the measured and predicted SOC trends if only the upper centimeters of the soils are observed. Overall, the results show that EO is a powerful tool for deriving topsoil SOC changes, while these do not correspond to the total SOC stocks in all cases.

### 4.6 Impact, Limitations, and Further Developments

Carbon removal from the atmosphere with C sequestration in soils can result in negative greenhouse gas emissions in agriculture and is developing as a promising business model to support efforts for climate change mitigation (Don et al. 2024; European Commission 2022). The monitoring of SOC in carbon farming programs has been outlined as a major challenge for success and to avoid greenwashing (Paul et al. 2023). The transaction costs for measuring SOC changes are often too high and jeopardize the establishment of carbon farming on a large scale (Don et al. 2024). Our results show that the integration of remote sensing data is a promising option to reduce the cost of SOC monitoring and generate high‐resolution information on cropland SOC dynamics beyond static SOC maps. Based on the comprehensive network of 100 LTM sites, we were able to provide a detailed verification of the predicted SOC trends and changes (Vaudour et al. 2022). These are the first steps in creating an integrated high‐resolution soil monitoring system that directly profits from the exploding availability of EO data (Paustian et al. 2019; Smith et al. 2020).

SRC reduce the satellite signal to bare soil observations and provide direct information on the soil state. Our findings demonstrate that they contain valuable information on SOC changes and should be a central part of spatiotemporal soil models. An increasing number of bare soil observations (Figure 3) offer the opportunity to further improve the signal‐to‐noise ratio and model accuracy (Figure 5). However, multiple challenges regarding the prediction and validation of SOC trends remain: Even though we had access to comprehensive soil monitoring data with seven measurements from 1986 to 2022, we were not able to capture and describe nonlinear SOC trends if they occurred. Similar to Venter et al. (2021), we exclusively relied on linear models to predict and validate the long‐term SOC trends. It has been shown, however, that SOC trends can change over time and alternate between SOC gains and losses (Wiesmeier et al. 2019). To improve the accuracy and predict nonlinear trends, it would be therefore necessary to increase the temporal resolution of the model and soil monitoring data. Due to the limited availability of EO data in earlier years, we had to rely on relatively broad intervals (± 4 years) to generate the dynamic SRC. This issue could, however, significantly decrease in the future following the increasing number of satellites and bare soil observations.

Still, our results based on the repeated HDB samples show that the prediction of short‐term SOC changes based on EO data remains challenging and is currently not possible (Figure S4). These findings underline the necessity of an integrated soil monitoring program to detect SOC changes in carbon farming schemes with smaller time scales (e.g., five years). Important pillars for this can be EU‐wide (e.g., the LUCAS Soil Survey), and national (e.g., the BZE) soil inventories, supported by comprehensive parcel databases on cropland management such as the Land Parcel Identification System (LPIS). Process modeling can integrate soil management data such as organic fertilization, crop rotation, or tillage and improve the estimations of short‐term SOC changes. Stratified soil sampling programs, with strong support from remote sensing, can help to scale the results down to a field level. In summary, SRC can provide comprehensive data on cropland soils and present a great opportunity to improve the information on long‐term SOC trends. Due to its high spatial and temporal resolution and increasing availability, EO can hardly be substituted by other data sources and should therefore be integrated into cropland soil monitoring programs.

## Author Contributions

**Tom Broeg:** conceptualization, data curation, formal analysis, investigation, methodology, software, validation, visualization, writing – original draft. **Axel Don:** conceptualization, data curation, funding acquisition, investigation, methodology, resources, validation, writing – review and editing. **Martin Wiesmeier:** conceptualization, data curation, investigation, methodology, resources, validation, writing – review and editing. **Thomas Scholten:** conceptualization, investigation, methodology, supervision, validation, writing – review and editing. **Stefan Erasmi:** conceptualization, data curation, funding acquisition, investigation, methodology, project administration, resources, supervision, validation, writing – review and editing.

## Conflicts of Interest

The authors declare no conflicts of interest.

## Supporting information


**Figure S1.** (a) Temporal trends of the model accuracy (*R* ^2^, RMSE, CCC, and RPD), based on all repeated LTM samples. (b) Distributions of the prediction intervals; Quartiles are illustrated with colors.
**Figure S2.** Cross‐validated SOC predictions and regression lines (red) for the LTM data below 40 g kg^−1^, based on the seven sampling periods from 1986 to 2022. 1:1‐lines are shown in black (dashed).
**Figure S3.** Accuracy of the cross‐validated SOC predictions and regression lines (red) for the HDB data, based on the two sampling periods from 2001 to 2018. 1:1‐lines are shown in black (dashed).
**Figure S4.** Distribution of the measured and predicted short‐term SOC changes (g kg^−1^) (a). The results are based on the HDB samples that were resampled after 10 years. (b) Direct comparison between the predicted and measured SOC changes.
**Figure S5.** Measured and predicted correlation coefficients (Pearson *r*) (a), and SOC trends (g kg^−1^ year^−1^) (b), grouped by the main soil texture of the LTM sites. *n* = 47/30/13/10 for loam/sand/silt/clay.
**Figure S6.** Map of the predicted cropland SOC uncertainty in 2021 (g kg^−1^). Map lines delineate study areas and do not necessarily depict accepted national boundaries.
**Figure S7.** Example of the SOC changes (g kg^−1^), calculated as the difference between the initial prediction in 1986 and the subsequent predictions from 1991 to 2021. Gains are displayed in green and losses in red. Map lines delineate study areas and do not necessarily depict accepted national boundaries.

## Data Availability

The data that support the findings of this study are openly available in Zenodo at https://doi.org/10.5281/zenodo.14191435. The soil data from the German Agricultural Soil Inventory (BZE‐LW) are available from OpenAgrar at https://doi.org/10.3220/DATA20200203151139.
