# Supplementary material for: Spatiotemporal Monitoring of Cropland Soil Organic Carbon Changes From Space
Source: Glob Chang Biol. 2024 Dec 9;30(12):e17608. doi: 10.1111/gcb.17608 (PMC11626691; doi:10.1111/gcb.17608)
Supplement: Supplementary file 1 — Figure S1. (a) Temporal trends of the model accuracy (R 2, RMSE, CCC, and RPD), based on all repeated LTM samples. (b) Distributions of the prediction intervals; Quartiles are illustrated with colors. Figure S2. Cross‐validated SOC predictions and regression lines (red) for the LTM data below 40 g kg−1, based on the seven sampling periods from 1986 to 2022. 1:1‐lines are shown in black (dashed). Figure S3. Accuracy of the cross‐validated SOC predictions and regression lines (red) for the HDB data, based on the two sampling periods from 2001 to 2018. 1:1‐lines are shown in black (dashed). Figure S4. Distribution of the measured and predicted short‐term SOC changes (g kg−1) (a). The results are based on the HDB samples that were resampled after 10 years. (b) Direct comparison between the predicted and measured SOC changes. Figure S5. Measured and predicted correlation coefficients (Pearson r) (a), and SOC trends (g kg−1 year−1) (b), grouped by the main soil texture of the LTM sites. n = 47/30/13/10 for loam/sand/silt/clay. Figure S6. Map of the predicted cropland SOC uncertainty in 2021 (g kg−1). Map lines delineate study areas and do not necessarily depict accepted national boundaries. Figure S7. Example of the SOC changes (g kg−1), calculated as the difference between the initial prediction in 1986 and the subsequent predictions from 1991 to 2021. Gains are displayed in green and losses in red. Map lines delineate study areas and do not necessarily depict accepted national boundaries. [file GCB-30-e17608-s001.docx]

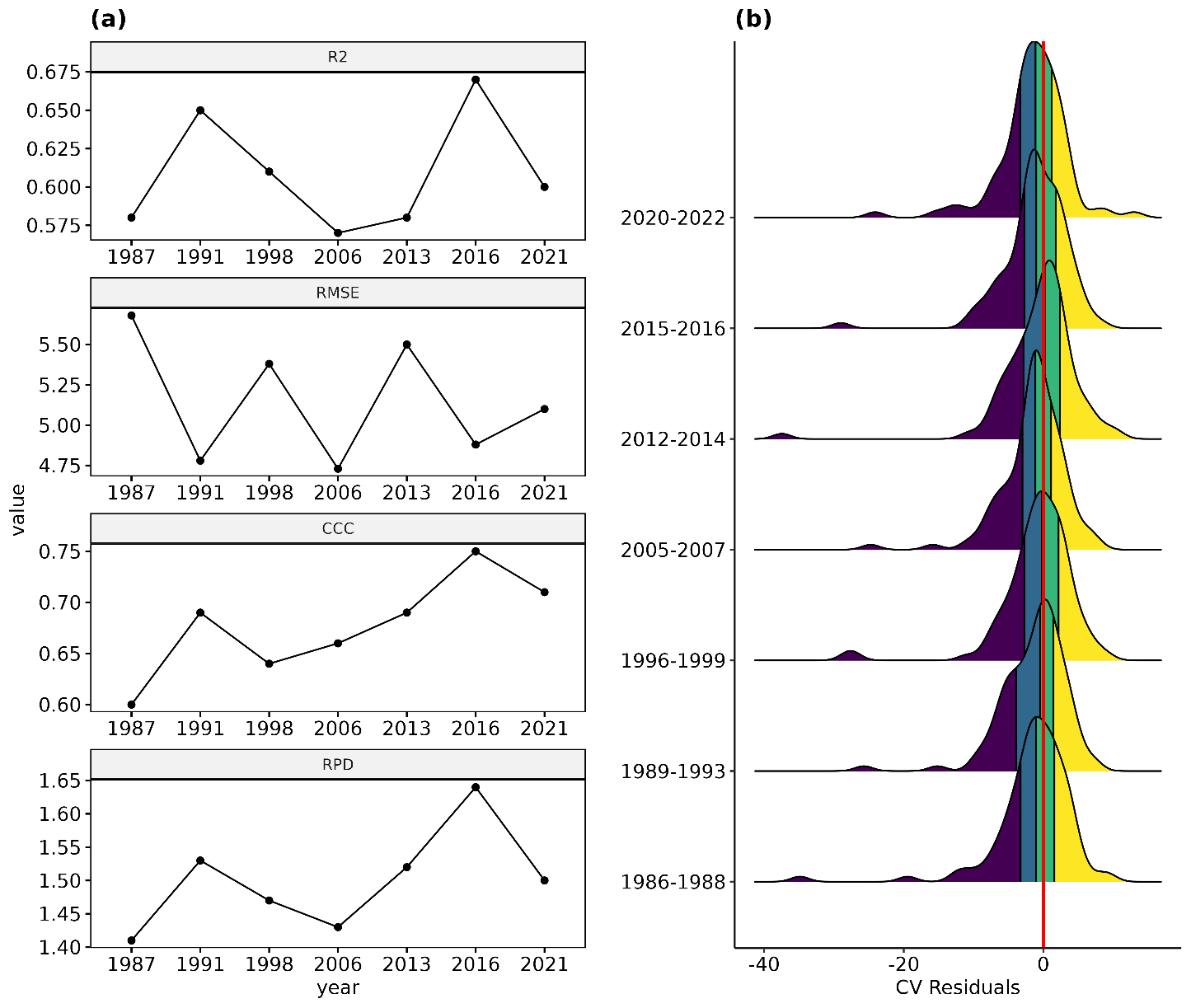


**Figure S1** | (a) Temporal trends of the model accuracy (R^2^, RMSE, CCC, and RPD), based on all repeated LTM samples. (b) Distributions of the prediction intervals; Quartiles are illustrated with colors.


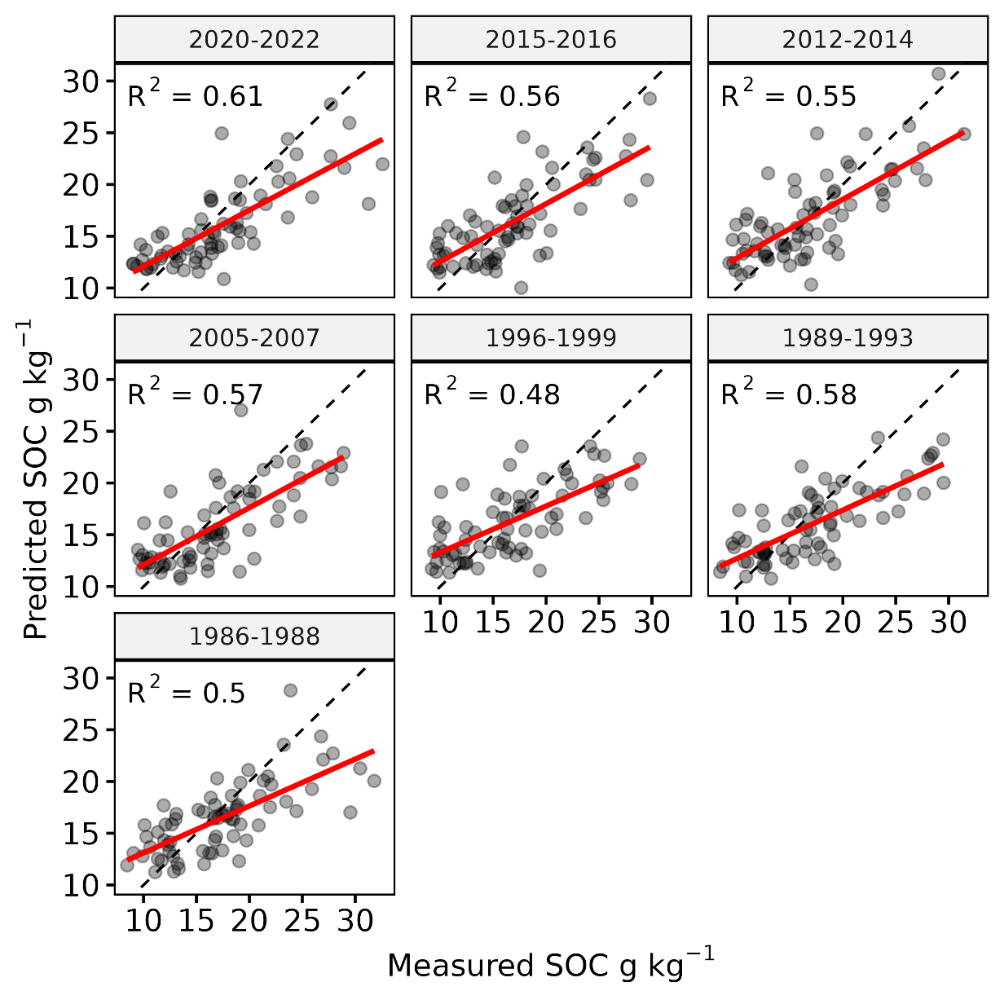


**Figure S2** | Cross-validated SOC predictions and regression lines (red) for the LTM data below 40 g kg^-1^, based on the seven sampling periods from 1986 to 2022. 1:1-lines are shown in black (dashed).


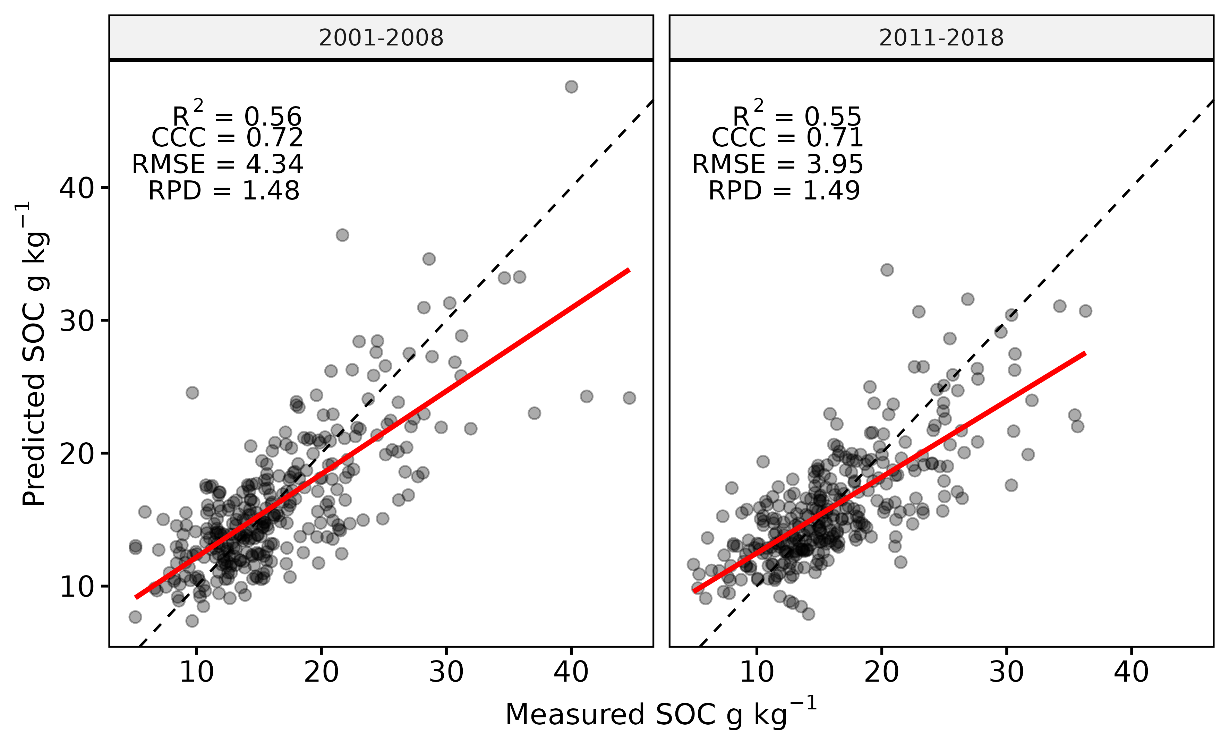


**Figure S3** | Accuracy of the cross-validated SOC predictions and regression lines (red) for the HDB data, based on the two sampling periods from 2001 to 2018. 1:1-lines are shown in black (dashed).


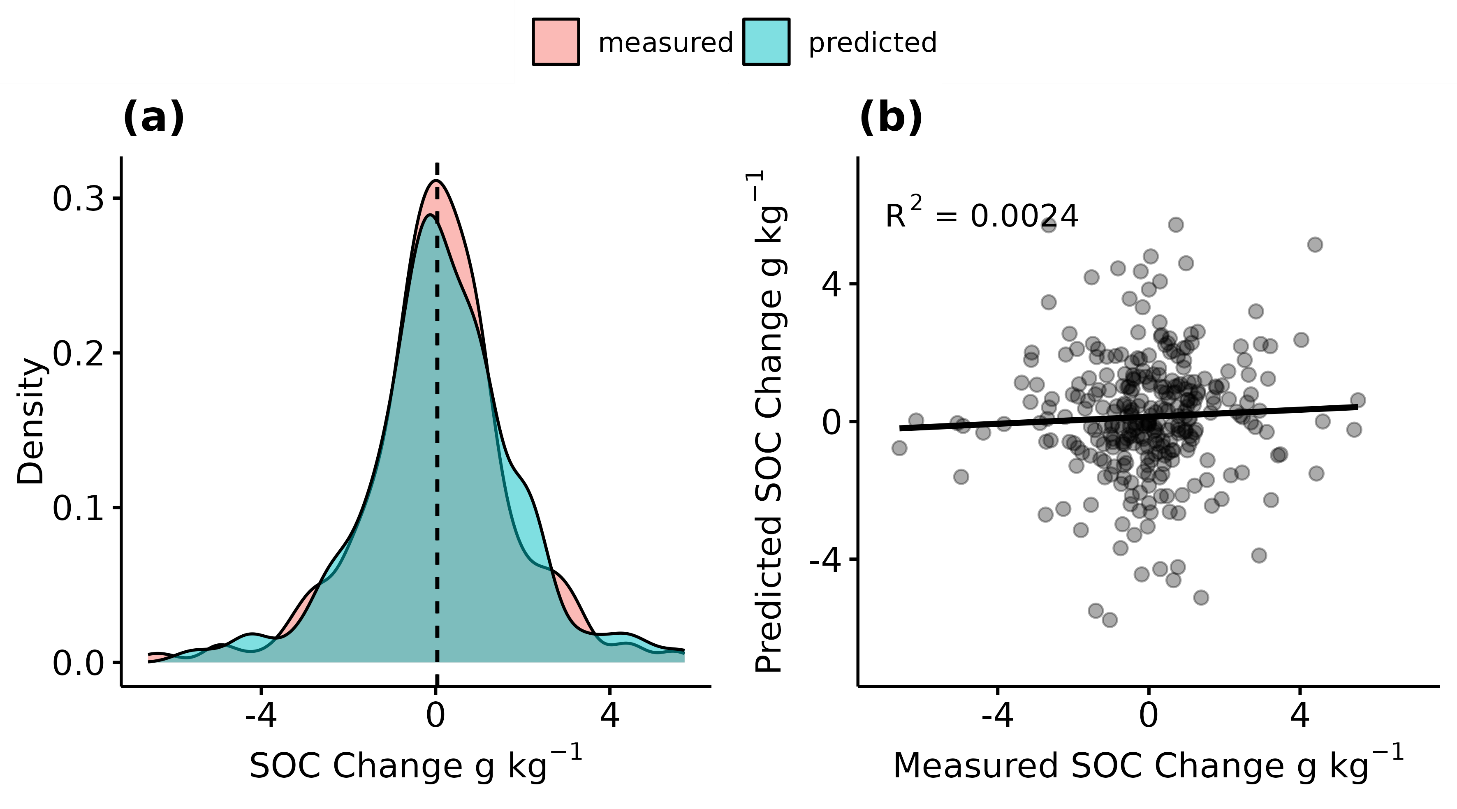


**Figure S4** | Distribution of the measured and predicted short-term SOC changes (g kg^-1^) (a). The results are based on the HDB samples that were resampled after ten years. (b) Direct comparison between the predicted and measured SOC changes.


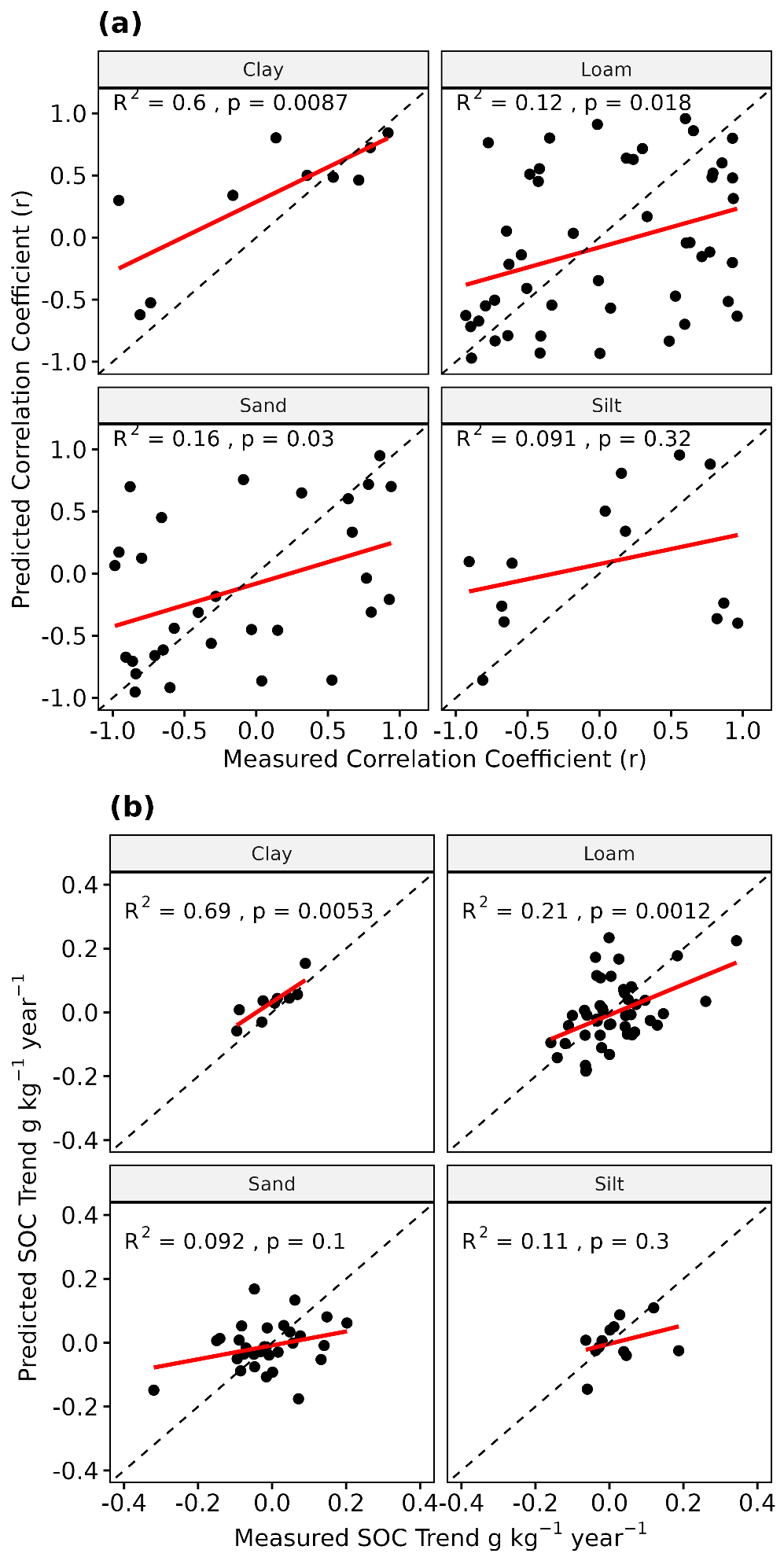


**Figure S5** | Measured and predicted correlation coefficients (Pearson r) (a), and SOC trends (g kg^-1^ year^-1^) (b), grouped by the main soil texture of the LTM sites. n = 47/30/13/10 for loam/sand/silt/clay.


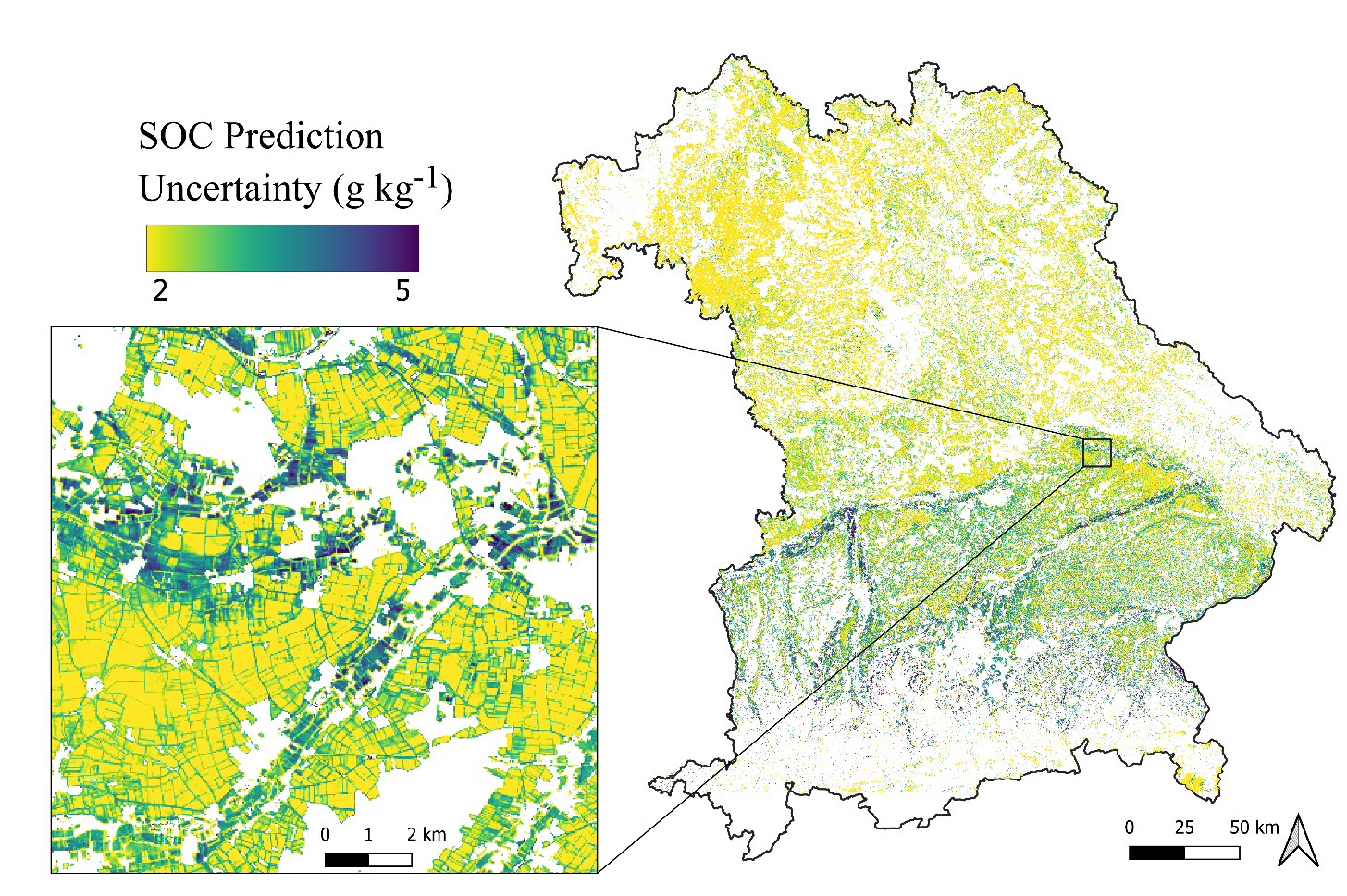


**Figure S6** | Map of the predicted cropland SOC uncertainty in 2021 (g kg^-1^). Map lines delineate study areas and do not necessarily depict accepted national boundaries.


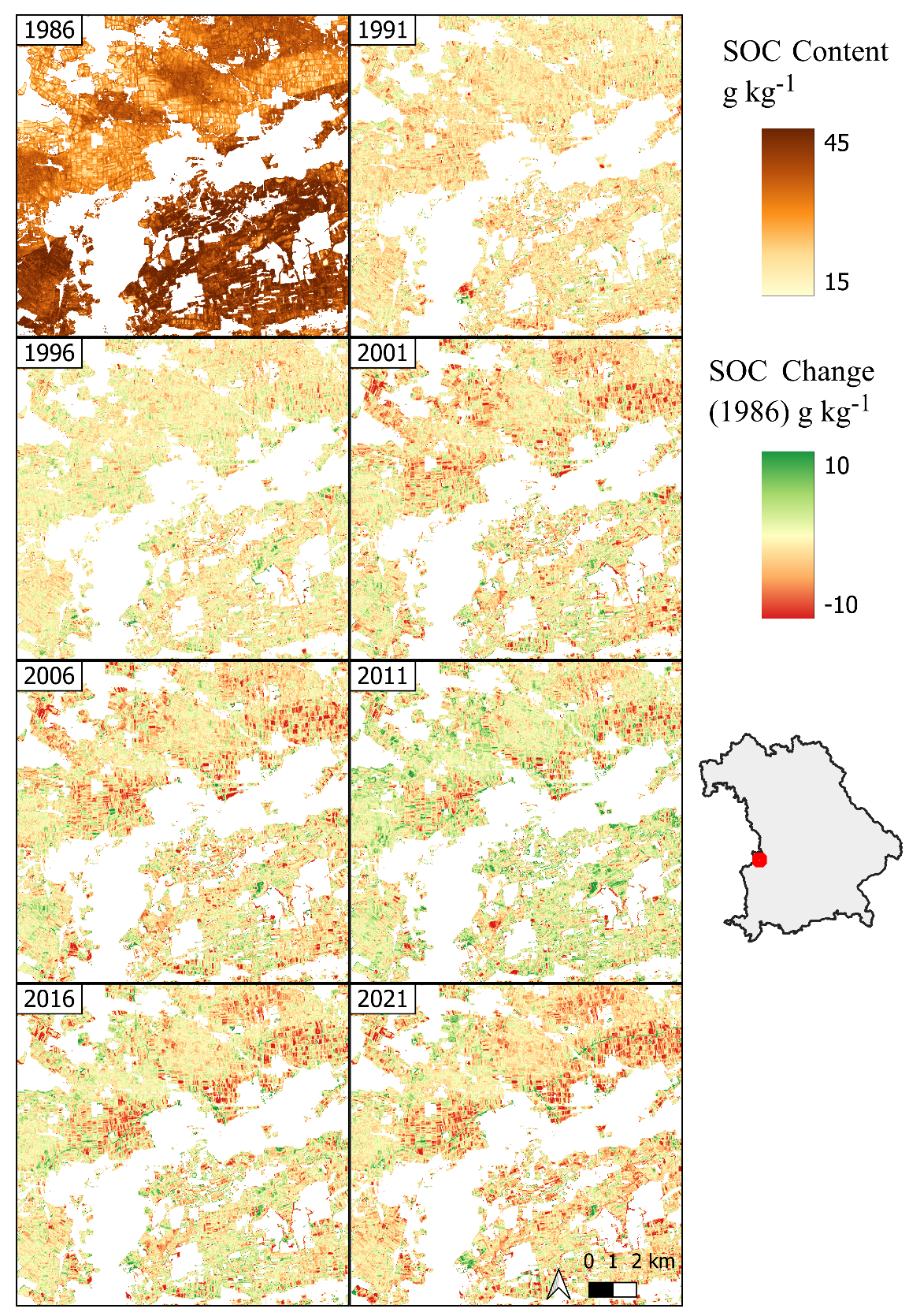


**Figure S7** | Example of the SOC changes (g kg-^1^), calculated as the difference between the initial prediction in 1986 and the subsequent predictions from 1991 to 2021. Gains are displayed in green and losses in red. Map lines delineate study areas and do not necessarily depict accepted national boundaries.
